# Integrative Taxonomy of Southeast Asian Snail-Eating Turtles (Geoemydidae: *Malayemys*) Reveals a New Species and Mitochondrial Introgression

**DOI:** 10.1371/journal.pone.0153108

**Published:** 2016-04-06

**Authors:** Flora Ihlow, Melita Vamberger, Morris Flecks, Timo Hartmann, Michael Cota, Sunchai Makchai, Pratheep Meewattana, Jeffrey E. Dawson, Long Kheng, Dennis Rödder, Uwe Fritz

**Affiliations:** 1 Herpetology Section, Zoologisches Forschungsmuseum Alexander Koenig, Bonn, Germany; 2 Museum of Zoology, Senckenberg Dresden, Dresden, Germany; 3 Thailand Natural History Museum, National Science Museum, Khlong Luang, Pathum Thani, Thailand; 4 Phranakhon Rajabhat University, Bang Khen, Bangkok, Thailand; 5 Charles H. Hoessle Herpetarium, Saint Louis Zoo, St. Louis, Missouri, United States of America; 6 General Department of Administration for Nature Conservation and Protection, Ministry of Environment, Chamkar Mon, Phnom Penh, Cambodia; University of Illinois at Urbana-Champaign, UNITED STATES

## Abstract

Based on an integrative taxonomic approach, we examine the differentiation of Southeast Asian snail-eating turtles using information from 1863 bp of mitochondrial DNA, 12 microsatellite loci, morphology and a correlative species distribution model. Our analyses reveal three genetically distinct groups with limited mitochondrial introgression in one group. All three groups exhibit distinct nuclear gene pools and distinct morphology. Two of these groups correspond to the previously recognized species *Malayemys macrocephala* (Chao Phraya Basin) and *M*. *subtrijuga* (Lower Mekong Basin). The third and genetically most divergent group from the Khorat Basin represents a previously unrecognized species, which is described herein. Although *Malayemys* are extensively traded and used for religious release, only few studied turtles appear to be translocated by humans. Historic fluctuations in potential distributions were assessed using species distribution models (SDMs). The Last Glacial Maximum (LGM) projection of the predictive SDMs suggests two distinct glacial distribution ranges, implying that the divergence of *M*. *macrocephala* and *M*. *subtrijuga* occurred in allopatry and was triggered by Pleistocene climate fluctuations. Only the projection derived from the global circulation model MIROC reveals a distinct third glacial distribution range for the newly discovered *Malayemys* species.

## Introduction

**Fig 1:**
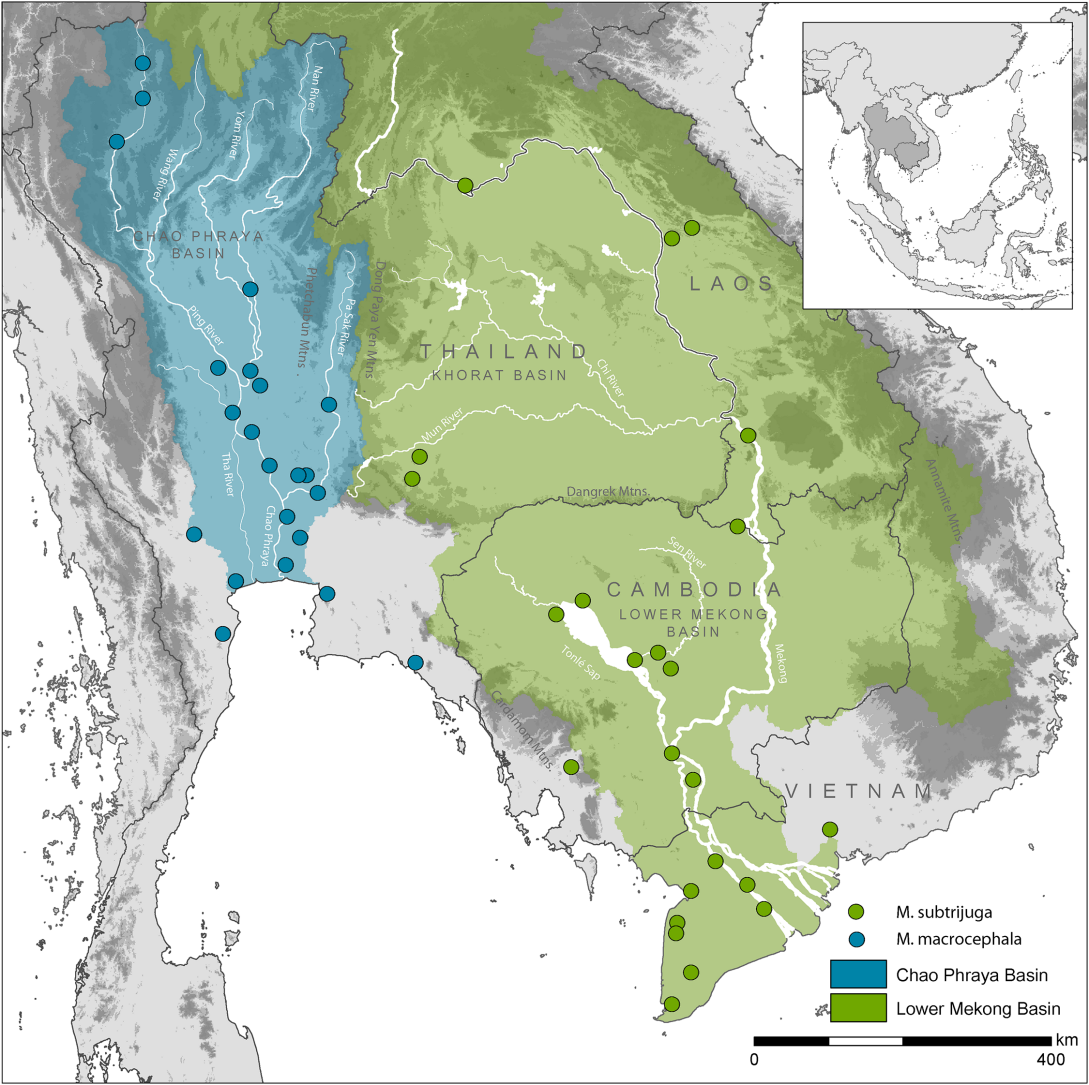
Geographic distribution of *Malayemys macrocephala* and *Malayemys subtrijuga* across most of Thailand, Laos, Cambodia and Vietnam as proposed by [1]. Locality records represent museum and literature records taken from [1].

Snail-eating Turtles of the genus *Malayemys* inhabit a variety of natural and anthropogenic freshwater habitats across the lowlands of Southeast Asia [1]. The genus was long considered monotypic [2–4], but recently, *Malayemys subtrijuga* (Schlegel & Müller, 1845) was restricted to populations from the eastern part of the distribution range, whereas the western populations were allocated to the resurrected species *Malayemys macrocephala* (Gray, 1859) [1,5,6] (Fig 1). However, this taxonomic assignment was solely based on morphological traits of preserved material [1,6]. The application of molecular approaches has become a standard tool in taxonomy, which can facilitate the detection of cryptic diversity (e.g. [7]) and has led to multiple revisions and descriptions of new taxa, particularly in turtles (e.g., [8–17]). Aquatic turtles can be taxonomically challenging, as they often exhibit high levels of genetic diversity coupled with conservative morphology [17–20]. Thus, a combination of different lines of evidence is crucial to elucidate the taxonomy of such groups with confidence.

Correlative species distribution models (SDMs) are widely used to estimate potential distributions of species across space and time [21]. Projections of potential distributions onto paleoclimatic conditions combined with phylogenetic patterns allow for quantification of the impacts of past climate changes on present day distributions [22] and can help to explain past range dynamics, diversity patterns and evolutionary processes [23].

Herein, we re-examine the taxonomy of *Malayemys* by combining phylogenetic analyses of two mitochondrial genes and analyses of twelve microsatellite loci with multivariate morphological methods. Further, we assess the present potential distribution of *Malayemys* and subsequently project it onto paleoclimatic conditions of the last glacial maximum using two global circulation models (CCSM, MIROC), to explore potential barriers to dispersal, past range dynamics and possible refugia. Two alternative hypotheses were tested: (*i*) the two presently recognized species represent a single, morphologically variable but genetically uniform species with a wide distribution range, or (*ii*) climatic fluctuations and recent geomorphological events led to allopatric divergence within *Malayemys*, yielding multiple distinct species with smaller ranges.

## Materials and Methods

### Ethics statement

Field work in Cambodia was carried out with permission of the General Department of Administration for Nature Conservation and Protection (GDANCP) of the Ministry of Environment (MoE), Cambodia (#178 covering all procedures of this project) while field research in Thailand was conducted in close collaboration with the Thailand Natural History Museum and the Phranakhon Rajabhat University in accordance to national law requiring no separate permits. This research was in accordance with the ethical guidelines on animal handling as required by the Zoologisches Forschungsmuseum Alexander Koenig and the Rheinische-Friedrich-Wilhelms-Universität, Bonn, Germany. Export of scientific samples was approved by the Cambodian MoE which provided the required certificate (#811 MoE) while samples and specimens from Thailand remain the property of the Thailand Natural History Museum and were placed at our disposal on loan to facilitate this study.

### Protection status

To our knowledge *Malayemys subtrijuga* is presently not protected in Cambodia, however regulations to hunting and trade of all aquatic wild animals exist (Law No 33 and 1563, Department of Fisheries). In Thailand, *M*. *subtrijuga* is fully protected from all forms of exploitation under the Thai Wild Animals Reservation and Protection Act, B.E. 2535, by listing in Schedule 2 (2 special) while *M*. *macrocephala* has not been assessed yet.

### Sampling

**Fig 2:**
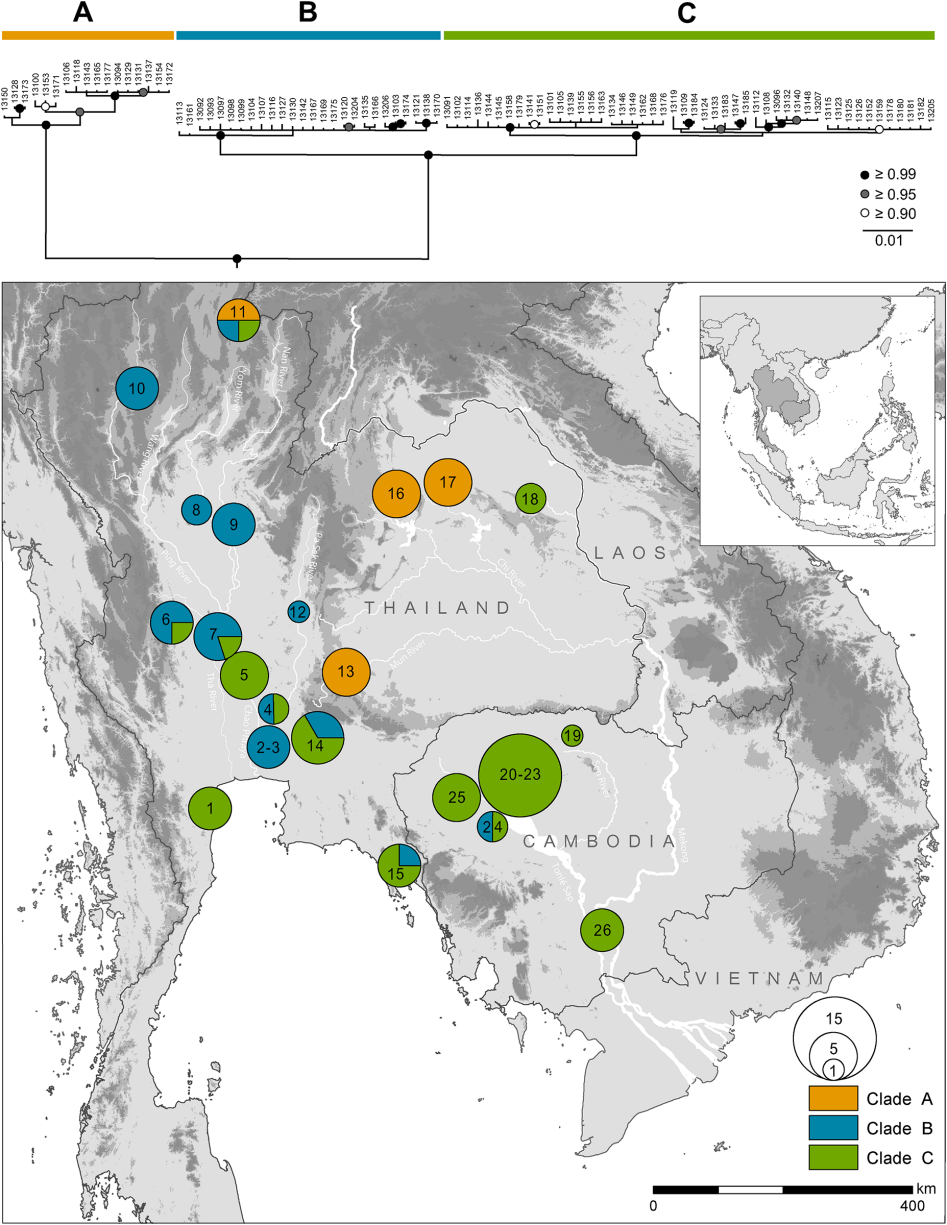
Distribution of mitochondrial clades of *Malayemys* in Thailand and Cambodia. Circle size is proportional to number of specimens per locality; coloration according to clades. The consensus tree from Bayesian inference is based on 1863 bp of mitochondrial DNA and shows the phylogenetic relationships of clades. Outgroup (*Heosemys annandalii*) removed for clarity. Nodes with Bayesian posterior probabilities (BPP) of 1 are indicated by solid black circles, BPP ≥ 0.95 by grey circles, and ≥ 0.9 by empty circles.

A total of 92 turtles, collected from 26 different localities across Thailand and Cambodia were examined (Fig 2; Table 1). Sampling sites were situated in three drainage systems, namely the Chao Phraya Basin (Thailand), the Khorat Basin (north-eastern Thailand) and the Tonlé Sap Lake (part of the Lower Mekong Basin, central Cambodia) and cover major parts of the distribution range of *Malayemys* (Figs 1 and 2; Table 1). All study sites are located outside of designated protected areas, except for the Prek Toal area of the Tonlé Sap Biosphere Reserve, Cambodia. While in Thailand live turtles were primarily obtained from resident fishermen and local markets, the majority of turtles from Cambodia were caught during our own field research and supplemented by few specimens obtained from local markets. For genetic analyses of living turtles, samples of either 100 μl of blood (drawn from the coccygeal vein) or, in few cases, a small section of tissue clipped from the tail tip was collected by FI and preserved in 900 μl analytical ethanol (98%). Tissue sample size varied between 2-3mm (depending on the size of the turtle) and was collected from the distal-most tip of the tail. The collection of blood and tail tip samples represent non-lethal standard methods commonly applied to turtles and no deleterious effects were observed in response to the procedure. Additional samples were acquired by extracting muscle or bone tissue from freshly dead (road kills) or preserved specimens in the Natural History Museum, Pathum Thani, Thailand. Except for four specimens retained as vouchers, living turtles were released at or near the site of capture after data collection. Voucher specimens constituting the type series were euthanized by intravenous injection of pentobarbital sodium, fixed in 90% ethanol and deposited at the following collections: Natural History Museum, Pathum Thani, Thailand (THNHM), catalogue number: THNHM 25816 and THNHM 25999; Zoologisches Forschungsmuseum Alexander Koenig, Bonn, Germany (ZFMK) catalogue number: ZFMK 97198 and the Museum für Tierkunde, Senckenberg Dresden, Germany (MTD) catalogue number: MTD 49150. These specimens are publicly deposited and accessible by other researchers in the three mentioned collections, constituting permanent repositories.

**Table 1:** Taxon sampling for genetic analyses. Geographic sampling localities, isolate numbers (MTD T), and respective GenBank accession numbers.

Locality	Pop. ID	Coordinates (WGS84)	MTD T	ncDNA cluster	mtDNA clade	Cyt b	ND4
**Thailand**:							
Petchaburi	1	13.10645°N, 99.94895°E	13091	B	C	-	KU848669
Petchaburi	1	13.10645°N, 99.94895°E	13141	B	C	KU848624	KU848713
Petchaburi	1	13.10645°N, 99.94895°E	13151	B	C	KU848633	KU848723
Petchaburi	1	13.10645°N, 99.94895°E	13168	B	C	KU848647	KU848737
Pathum Thani, Mueang Dist.	2	14.04790°N, 100.71575°E	13092	B	B	KU848580	KU848670
Pathum Thani, Mueang Dist.	2	14.04790°N, 100.71575°E	13130	B	B	KU848613	KU848702
Pathum Thani	3	13.98840°N, 100.55634°E	13157	B	-	-	-
Ayutthaya	4	14.33818°N, 100.57956°E	13099	B	B	KU848586	KU848676
Ayutthaya	4	14.33818°N, 100.57956°E	13105	B	C	KU848592	KU848681
Tha Chang	5	14.78262°N, 100.40274°E	13101	B	C	KU848588	KU848678
Tha Chang	5	14.78262°N, 100.40274°E	13139	B	C	KU848622	KU848711
Tha Chang	5	14.78262°N, 100.40274°E	13146	B	C	KU848628	KU848718
Tha Chang	5	14.78262°N, 100.40274°E	13162	B	C	KU848642	KU848732
Tha Chang	5	14.78262°N, 100.40274°E	13176	B	C	KU848655	KU848745
Uthai Thani	6	15.49055°N, 99.54609°E	13097	B	B	KU848584	KU848674
Uthai Thani	6	15.49055°N, 99.54609°E	13135	B	B	KU848618	KU848707
Uthai Thani	6	15.49055°N, 99.54609°E	13138	B	B	KU848621	KU848710
Uthai Thani	6	15.49055°N, 99.54609°E	13144	B	C	KU848626	KU848716
Tap Than	7	15.48752°N, 99.87959°E	13113	B	B	KU848598	KU848687
Tap Than	7	15.48752°N, 99.87959°E	13121	B	B	KU848605	KU848694
Tap Than	7	15.48752°N, 99.87959°E	13156	B	C	KU848638	KU848728
Tap Than	7	15.48752°N, 99.87959°E	13170	B	B	KU848649	KU848739
Tap Than	7	15.48752°N, 99.87959°E	13175	B	B	KU848654	KU848744
Sukothai	8	17.01198°N, 99.82058°E	13167	B	B	KU848646	KU848736
Sukothai	8	17.01198°N, 99.82058°E	13169	B	B	KU848648	KU848738
Phitsanulok	9	16.82438°N, 100.26191°E	13104	B	B	KU848591	KU848680
Phitsanulok	9	16.82438°N, 100.26191°E	13107	B	B	KU848594	KU848683
Phitsanulok	9	16.82438°N, 100.26191°E	13116	B	C	KU848601	KU848690
Phitsanulok	9	16.82438°N, 100.26191°E	13166	B	B	KU848645	KU848735
Lamphun	10	18.59711°N, 99.00055°E	13103	B	B	KU848590	KU848679
Lamphun	10	18.59711°N, 99.00055°E	13174	B	B	KU848653	KU848742
Lamphun	10	18.59711°N, 99.00055°E	13204	B	B	KU848665	KU848755
Lamphun	10	18.59711°N, 99.00055°E	13206	B	B	KU848667	KU848757
Chiang Kham	11	19.52470°N, 100.30430°E	13098	B	B	KU848585	KU848675
Chiang Kham	11	19.52470°N, 100.30430°E	13129	B	A	KU848612	KU848701
Chiang Kham	11	19.52470°N, 100.30430°E	13134	B	C	KU848617	KU848706
Chiang Kham	11	19.52470°N, 100.30430°E	13154	B	A	KU848636	KU848726
Wichinburi	12	15.68251°N, 101.10829°E	13127	B	B	KU848610	KU848699
Sikhio	13	14.88968°N, 101.72565°E	13094	A	A	KU848582	KU848672
Sikhio	13	14.88968°N, 101.72565°E	13131	A	A	KU848614	KU848703
Sikhio	13	14.88968°N, 101.72565°E	13137	A	A	KU848620	KU848709
Sikhio	13	14.88968°N, 101.72565°E	13153	A	A	KU848635	KU848725
Sikhio	13	14.88968°N, 101.72565°E	13172	A	A	KU848651	KU848741
Patchinburi	14	14.02195°N, 101.37596°E	13102	B	C	KU848589	-
Patchinburi	14	14.02195°N, 101.37596°E	13120	B	B	KU848604	KU848693
Patchinburi	14	14.02195°N, 101.37596°E	13142	B	B	-	KU848714
Patchinburi	14	14.02195°N, 101.37596°E	13149	B	C	KU848631	KU848721
Patchinburi	14	14.02195°N, 101.37596°E	13155	B	C	KU848637	KU848727
Patchinburi	14	14.02195°N, 101.37596°E	13179	B	C	KU848658	KU848748
Trat	15	12.38329°N, 102.65776°E	13114	B	C	KU848599	KU848688
Trat	15	12.38329°N, 102.65776°E	13145	B	C	KU848627	KU848717
Trat	15	12.38329°N, 102.65776°E	13158	B	C	KU848639	KU848729
Trat	15	12.38329°N, 102.65776°E	13161	B	B	KU848641	KU848731
Nong Bua	16	17.22455° N, 102.42678° E	13106	A	A	KU848593	KU848682
Nong Bua	16	17.22455° N, 102.42678° E	13118	A	A	KU848602	KU848691
Nong Bua	16	17.22455° N, 102.42678° E	13143	A	A	KU848625	KU848715
Nong Bua	16	17.22455° N, 102.42678° E	13165	A	A	KU848644	KU848734
Nong Bua	16	17.22455° N, 102.42678° E	13177	A	A	KU848656	KU848746
Udon Thani	17	17.36555°N, 102.81427°E	13100	A	A	KU848587	KU848677
Udon Thani	17	17.36555°N, 102.81427°E	13128	A	A	KU848611	KU848700
Udon Thani	17	17.36555°N, 102.81427°E	13150	A	A	KU848632	KU848722
Udon Thani	17	17.36555°N, 102.81427°E	13171	A	A	KU848650	KU848740
Udon Thani	17	17.36555°N, 102.81427°E	13173	A	A	KU848652	KU848742
Sakhon Nakhon	18	17.15515°N, 104.13543°E	13119	B	C	KU848603	KU848692
Sakhon Nakhon	18	17.15515°N, 104.13543°E	13136	B	C	KU848619	KU848708
**Cambodia**:							
Preah Vihear	19	14.06039°N, 104.67576°E	13207	C	C	KU848668	KU848758
Prek Toal	20	13.23234°N, 103.64497°E	13096	C	C	KU848583	KU848673
Prek Toal	20	13.23234°N, 103.64497°E	13115	C	C	KU848600	KU848689
Prek Toal	20	13.23234°N, 103.64497°E	13124	C	C	KU848607	KU848696
Prek Toal	20	13.23234°N, 103.64497°E	13125	C	C	KU848608	KU848697
Prek Toal	20	13.23234°N, 103.64497°E	13140	C	C	KU848623	KU848712
Prek Toal	20	13.23234°N, 103.64497°E	13147	C	C	KU848629	KU848719
Prek Toal	20	13.23234°N, 103.64497°E	13148	C	C	KU848630	KU848720
Prek Toal	20	13.23234°N, 103.64497°E	13152	C	C	KU848634	KU848724
Prek Toal	20	13.23234°N, 103.64497°E	13159	C	C	KU848640	KU848730
Prek Toal	20	13.23234°N, 103.64497°E	13178	C	C	KU848657	KU848747
Prek Toal	21	13.23322°N, 103.65009°E	13108	C	C	KU848595	KU848684
Prek Toal	21	13.23322°N, 103.65009°E	13126	C	C	KU848609	KU848698
Prek Toal	22	13.23647°N, 103.64030°E	13109	C	C	KU848596	KU848685
Prek Toal	22	13.23647°N, 103.64030°E	13112	C	C	KU848597	KU848686
Prek Toal	23	13.23149°N, 103.63761°E	13133	C	C	KU848616	KU848705
Tonlé Sap Lake	24	13.02718°N, 103.64497°E	13093	B	B	KU848581	KU848671
Tonlé Sap Lake	24	13.02718°N, 103.64497°E	13163	C	C	KU848643	KU848733
Tonlé Sap Lake, Battambang	25	13.31839°N, 103.40611°E	13123	C	C	KU848606	KU848695
Tonlé Sap Lake, Battambang	25	13.31839°N, 103.40611°E	13132	C	C	KU848615	KU848704
Tonlé Sap Lake, Battambang	25	13.31839°N, 103.40611°E	13180	C	C	KU848659	KU848749
Tonlé Sap Lake, Battambang	25	13.31839°N, 103.40611°E	13181	C	C	KU848660	KU848750
Tonlé Sap Lake, Battambang	25	13.31839°N, 103.40611°E	13205	C	C	KU848666	KU848756
Tonlé Sap Lake, Phnom Penh	26	11.52386°N, 105.07048°E	13182	C	C	KU848661	KU848751
Tonlé Sap Lake, Phnom Penh	26	11.52386°N, 105.07048°E	13183	C	C	KU848662	KU848752
Tonlé Sap Lake, Phnom Penh	26	11.52386°N, 105.07048°E	13184	C	C	KU848663	KU848753
Tonlé Sap Lake, Phnom Penh	26	11.52386°N, 105.07048°E	13185	C	C	KU848664	KU848754

### Laboratory procedure and phylogenetic analyses of mitochondrial DNA

Two mitochondrial DNA fragments, that have been successfully used for phylogenetic and phylogeographic purposes in geoemydid turtles (e.g. [8,9,18,24]) were utilized, namely: the nearly complete cytochrome *b* gene (cyt *b*) plus adjacent DNA coding for tRNA-Thr and the 3' half of the NADH dehydrogenase subunit 4 gene (ND4) plus adjacent DNA coding for tRNAs. Total genomic DNA of fresh tissue and blood samples was extracted using the innuPREP DNA Mini Kit and the innuPREP Blood DNA Mini Kit (Analytik Jena AG, Jena, Germany), respectively. The mitochondrial DNA blocks were amplified using PCRs having a final volume of 25 μl. The reaction mix for both genes contained 1 unit *Taq* polymerase (Bioron, Ludwigshafen, Germany) with PCR buffer 10× including MgCl_2_, 0.2 mM of each dNTP (Thermo Scientific, St. Leon-Rot, Germany), 0.4 mM of each primer, ultrapure H_2_O, and 10–50 ng of total DNA. The cyt *b* fragment was amplified using the primer pair CytbG [8] and mt-f-na [25]. To amplify the ND4 fragment, the primer pair L-ND4 and H-Leu [9] was used. Thermocycling protocols are given in S1 Table. PCR products were purified using the ExoSAP-IT enzymatic clean-up (USB Europe GmbH, Staufen, Germany; modified protocol: 30 min at 37°C; 15 min at 80°C) and sequenced on an ABI 3730 Genetic Analyzer (Applied Biosystems, Foster City, CA, USA) using the BigDye Terminator v3.1 Cycle Sequencing Kit (Applied Biosystems) and either the primers CytbG, mt-C-For2, and mt-f-na for the cyt *b* fragment; or the primers L-ND4 and H-Leu for the ND4 fragment. Cycle sequencing reactions were purified using Sephadex^™^ (GE Healthcare, München, Germany) and 25 cycles were run with denaturing at 96°C for 10 s, annealing at 50°C for 5 s and elongation at 60°C for 4 min.

Cyt *b* and ND4 sequences were obtained from 89 and 90 samples, respectively (Table 1). These were checked using the original electropherograms and subsequently manually aligned in bioedit 7.0.5.2 [26]. Homologous sequences of *Heosemys annandalii* were downloaded from GenBank (accession number JF742646) and added as outgroup. The final concatenated dataset consisted of 1863 bp (1078 bp of cyt *b* plus adjacent tRNA and 785 bp of ND4 plus adjacent tRNAs) and was partitioned by gene and codon position. Partitions were tested with partitionfinder v1.1.1 [27], which also selects the best-fitting nucleotide substitution model using the Bayesian Information Criterion. Chosen models and partitions were HKY+G for the first codon positions of cyt *b* and ND4, and the tRNA; HKY+I for the second codon positions of cyt *b* and ND4; and HKY for the third codon positions of cyt *b* and ND4.

Phylogenetic trees were inferred with mrbayes 3.2.2 [28]. Model parameters were estimated separately for each of the three partitions by unlinking them. Four independent runs were performed with 10 million generations each, sampling every 1,000 trees. However, the analysis was stopped when the average standard deviation of split frequencies fell below 0.01. Results of the MCMC were summarized and the initial 1,000 trees of each run discarded as burn-in after checking for convergence and sufficient effective sample sizes in tracer v1.6 [29].

Relationships of DNA sequences were further assessed by building haplotype networks using a statistical parsimony approach as implemented in tCS 1.21 [30]. Networks were constructed separately for the DNA fragments containing the cyt *b* and ND4 genes. In addition, uncorrected *p*-distances were calculated for cyt *b* and ND4 using mega 6.06 [31] and the pairwise deletion option.

### Laboratory procedure and population genetic analyses of microsatellite data

Using primers developed for other species [32–38], 12 microsatellite loci were amplified in 92 samples of *Malayemys* (S2 Table). These loci turned out to be highly polymorphic.

Microsatellite DNA was amplified using individual PCRs for each locus or multiplex PCR (S2 Table), each in a final volume of 10 μl containing 0.5 units *Taq* polymerase (Bioron) together with the buffer recommended by the supplier, 0.375 mM of MgCl_2_ (Bioron), 0.2 mM of each dNTP (Thermo Scientific), 2 μg of bovine serum albumin (Thermo Scientific), 0.25 mM of each primer and 20–40 ng of total DNA. Cycling conditions were as follows: 35 cycles with denaturation at 94°C for 45 s, preceded by an initial denaturation step of 5 min, annealing at primer-specific temperature (S2 Table) for 60 s and extension at 72°C for 60 s, followed by final elongation of 10 min. PCR products were diluted with water in a ratio of 1:100. Fragment lengths were determined on an ABI 3730 Genetic Analyzer (Applied Biosystems) using the GeneScan–600 LIZ Size Standard (Applied Biosystems) and the software peak scanner 1.0 (Life Technologies, Carlsbad, CA).

The 12 loci were analysed with an unsupervised Bayesian clustering approach as implemented in structure 2.3.4 [39,40] using the admixture model and correlated allele frequencies. The advantage of such unsupervised analyses is that the software clusters the samples strictly according to the genetic information, but without any presumptions about population structuring (e.g. geographical distances, sampling sites). structure searches the data set for partitions which are, as far as possible, in Hardy-Weinberg equilibrium and linkage equilibrium. For all analyses, the upper bound for calculations was set arbitrarily to *K* = 10, and the most likely number of clusters (*K*) was determined using the Δ*K* method [41] with the software structure harvester [42]. Calculations were repeated 10 times for each *K* using a MCMC chain of 750,000 generations for each run, including a burn-in of 250,000 generations. Population structuring and individual admixture were visualized using the software distruct 1.1 [43]. Individuals below a threshold of 80% for cluster membership were treated as having mixed ancestries [44].

Diversity and divergence parameters were estimated for all three clusters with microsatellite data. For comparing the number and size of microsatellite alleles, a frequency table was produced using convert 1.31 [45]. Genetic differentiation between clusters was examined with arlequin 3.11 [46] using *F*_ST_ values and analyses of molecular variance (AMOVAs; 10,000 permutations). In addition, arlequin was used to estimate locus-specific observed (*H*_O_) and expected heterozygosities (*H*_E_). The software fstat 2.9.3.2 [47] was used for determining locus-specific excess or deficiency of heterozygotes as expressed by the inbreeding coefficient *F*_IS_ [48] and also the statistical significance of *F*_IS_. Finally, values for locus-specific allelic richness were calculated with the same software.

### Morphology

A total of 94 specimens of *Malayemys* was examined for 28 morphometric and 13 coloration-related characters (meristic *n* = 3, categorical *n* = 10). Metric characters of carapace and plastron were measured to the nearest mm by the same person (FI) using a digital calliper for straight-line characters and a measuring tape for curved measurements. The area of dark plastron pigmentation was assessed as percentage of the total plastron area using a colour threshold method in ImageJ [49]. The threshold was evaluated using a binary image and set to the value that captured the dark pigmentation completely. Subsequently, all images were processed with this threshold. The number of nasal stripes (NasS) and the shape of the infraorbital stripe (InfLorb) were determined following Brophy [6]. For the complete set of examined characters, see S1 Text. As turtles are known to be sexually dimorphic regarding their shell size and shape [50–54], all analyses were conducted for males and females separately. Sexes were determined according to tail morphology as described by Brophy [6]. To avoid size-dependent intercorrelation effects in the morphometric data, we calculated regression residuals on the log-transformed metric variables using straight carapace length as a covariable.

A principal component analysis (PCA) was conducted using mixed variables as implemented in the ade4 package [55] for cran R [56]. This method can handle quantitative and categorical variables [57], allowing for the inclusion of coloration data. Such categorical characters can be important to distinguish taxa, and therefore should not be neglected in multivariate analyses [58]. The first four principal components (PCs; with Eigenvalues > 1) were used for subsequent analyses to capture major parts of the total variation. Restriction to four PCs was necessary as a trade-off between the number of observations per group and dimensionality of the morphological space.

Subsequently, a multivariate kernel density estimation (KDE) was used to estimate the *n*-dimensional hypervolume of each of the different clades of *Malayemys* using the hypervolume package in R [59]. The KDE produces random points with a uniform distribution in morphospace defined by a minimum convex polytope enclosing the observations. For each species pair, we determined the total volume (union of both hypervolumes), the overlap (intersection of hypervolumes), and the unique part of each hypervolume. Furthermore, the Soerensen index based on the unique and shared parts was calculated as a measure of pairwise overlap in multidimensional morphological space.

### Species distribution models

The potential distribution of *Malayemys* was assessed through a correlative species distribution model using maxent version 3.3.3k [60,61], which performs comparatively well even with a restricted number of records [62]. A set of 29 genetically verified occurrence records was used to build the model. In addition, six uncorrelated bioclimatic variables with a spatial resolution of 2.5 arc minutes were obtained from WorldClim (www.worldclim.org): annual mean temperature and precipitation (bio 1, bio 12), mean temperature of the warmest quarter (bio 10), mean temperature of the coldest quarter (bio 11), and precipitation of the wettest and driest quarter (bio 16, Bio 17). As training area, an area defined by a polygonal bounding box enclosing all species records was selected.

To predict the impact of historical climate fluctuations and related eustatic sea level changes on the distribution of *Malayemys*, we obtained two projections for the Last Glacial Maximum (LGM, 21,000 years ago) from global circulation models (GCM) through the Paleoclimate Modelling Intercomparison Project Phase II (PMIP2) [63], namely the Community Climate System Model (CCSM) [64], and the Model for Interdisciplinary Research on Climate (MIROC) [65]. Both GCMs were statistically down-scaled to a spatial resolution of 2.5 arc minutes using the delta method [66] and downloaded from www.worldclim.org.

In maxent, we applied a bootstrapping approach with 100 replicates splitting the occurrence data set randomly with 80% used for model training and 20% for model evaluation using the area under the receiver operating characteristic curve [67]. Only linear, quadratic and product features were allowed in order to restrict model complexity and to avoid spurious effects in response curves. The average projections across all 100 replicates were used for further processing, wherein the “balance training omission, predicted area and threshold value logistic threshold” were applied as non-fixed presence-absence threshold. Areas requiring extrapolation beyond the training area of the SDM were identified via multivariate environmental similarity surfaces (MESS) [68].

### Nomenclatural Acts

The electronic version of this article corresponds to the requirements of the amended International Code of Zoological Nomenclature [69]. Therefore, the new name contained herein is available under that Code from the electronic edition of this article. This published work and the nomenclatural act it contains have been registered in ZooBank, the online registration system for the ICZN. The ZooBank LSIDs (Life Science Identifiers) can be resolved and the associated information viewed through any standard web browser. The LSID for this publication is: urn:lsid:zoobank.org:pub:97BA99D1-13EA-4390-8A2D-2112CFA7D6B8. The electronic edition of this work was published in a journal with an ISSN, has been archived and is available from the following digital repositories: PubMed Central, LOCKSS.

## Results

### Mitochondrial phylogeny and geographic distribution of clades

Analysis of the concatenated mtDNA revealed three well-supported clades (Fig 2). With a single exception, Clade B comprises turtles from the Chao Phraya Basin. This clade is, with high support, sister to another clade (Clade C) containing mostly individuals from the Lower Mekong Basin. However, this clade also contains a number of individuals from the Chao Phraya Basin. The successive sister, again with high support, is Clade A, comprising mainly turtles from the Khorat Basin. Thus, the geographic distribution of Clade B and C roughly correspond to the ranges of the two previously recognized species: *Malayemys macrocephala* (Chao Phraya Basin) and *M*. *subtrijuga* (Lower Mekong Basin). Yet haplotypes of different clades were found in few instances at the same sites, especially in central and eastern Thailand (Fig 2).

While Clade B and C differed by uncorrected *p*-distances of 1.45% for cyt *b* and 1.09% for the ND4 fragment, Clade A was much more divergent. It differed from Clade B by 6.92% for cyt *b* and 5.30% for the ND4 fragment, and from Clade C by 6.81% for cyt *b* and 5.38% for the ND4 fragment (S5 Table).

For both mtDNA fragments, within-clade divergences were most pronounced for Clade A from the Khorat Basin (cyt *b*: 0.54%, ND4: 0.91%), while Clade C (cyt *b*: 0.43%, ND4: 0.21%) and Clade B (cyt *b*: 0.14%, ND4: 0.17%) showed lower values.

### Genetic structuring according to microsatellite data

**Fig 3:**
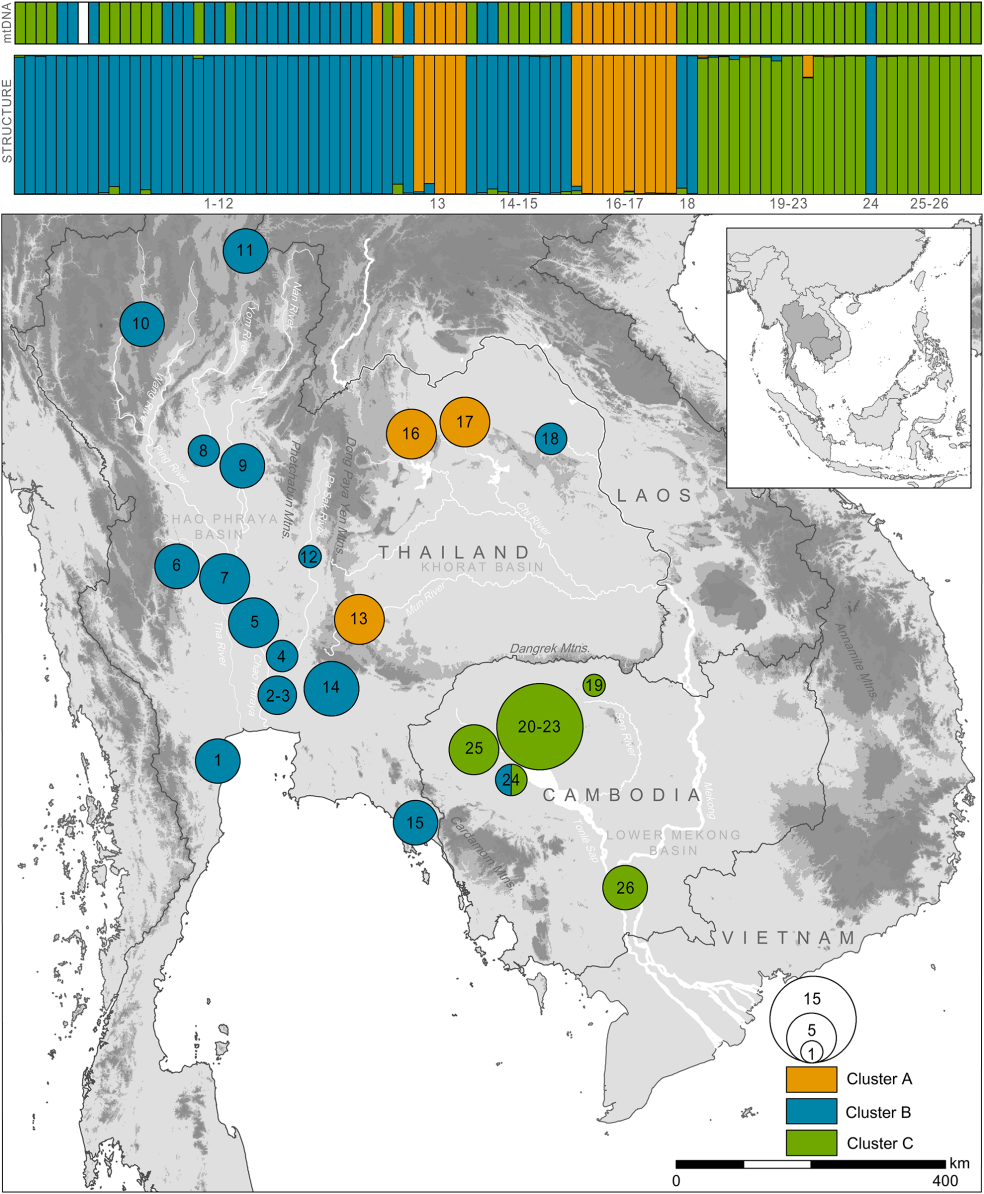
Genotypic structuring of 91 *Malayemys* specimens for *K* = 3 using 12 microsatellite loci (top). Shown is the structure run with the best probability value. Within each cluster an individual turtle is represented by a vertical segment that reflects its ancestry. Mixed ancestries are indicated by differently coloured sectors corresponding to inferred genetic percentages of the respective cluster. The mitochondrial lineage of each sample is shown above the structure diagrams. Colours of pooled sampling sites in the map correspond to structure clusters; slices represent turtles with conflicting cluster assignment (percentages). Numbers refer to population IDs (Table 1).

The Δ*K* method [41] revealed *K* = 3 as the optimal number of clusters. Cluster B corresponded to turtles primarily from the Chao Phraya Basin (*M*. *macrocephala*), Cluster A referred to turtles from the Khorat Basin, and Cluster C represented turtles from the Lower Mekong Basin (*M*. *subtrijuga*; Fig 3).

There was no evidence for gene flow across these three clusters. However, two misplaced individuals were identified in the second cluster (Fig 3; Site 18: Sakhon Nakhon), and a single one within the Lower Mekong cluster (Fig 3; Site 24: Tonlé Sap Lake). The latter misplacement is in concordance with the mitochondrial phylogeny.

However, individuals at two locations within the Lower Mekong Basin (Fig 3; Sites 18 and 24) were placed in the microsatellite cluster associated with *M*. *macrocephala* (Cluster B), rather than *M*. *subtrijuga* (Cluster C). Two of these individuals (Fig 3; at Site 18: Sakhon Nakhon) had discordant mitochondrial haplotypes corresponding to *M*. *subtrijuga* (Clade C). In addition, a single individual (Fig 3; Site 24: Tonlé Sap Lake) was identified as *M*. *macrocephala* by both microsatellite (Cluster B) and mitochondrial (Clade B) data.

### Diversity within and divergence among clusters

For the following diversity analyses, turtles were assigned to the three distinct clusters revealed by structure. Numbers of alleles per locus ranged from 2 to 22. Of a total of 134 alleles, 65 private alleles were found in only one of the three clusters (Table 2). With exception of the *F*_IS_ value, the highest genetic diversity indices were found in the Chao Phraya cluster (Cluster B). The highest *F*_ST_ value was found between the Khorat cluster (Cluster A) and the Lower Mekong cluster (Cluster A) (S3 Table, *F*_ST_ = 0.55) for microsatellites and the amova indicated that 43% of the observed global variation occurred among and 57% within clusters.

**Table 2:** Genetic diversity of clusters.

Microsatellites									mtDNA				
										Cyt *b*		ND4	
Cluster	*n*	*n*_*A*_	*n*_*Ā*_	*n*_*P*_	*AR*	*H*_*O*_	*H*_*E*_	*F*_*IS*_	Clade	*n*_*H*_	*n*_*HP*_	*n*_*H*_	*n*_*HP*_
Chao Phraya	26	78	6.5	19	5.28	0.42	0.45	0.05	C	29	22	18	11
Lower Mekong	51	103	8.6	36	6	0.5	0.58	0.138	B	14	10	10	7
Khorat	15	42	3.5	10	3.43	0.25	0.32	0.214	A	8	5	8	5

*n*, number of individuals; *n*_*A*_, number of alleles; *n*_*Ā*_, average number of alleles per locus; *n*_*P*_, number of private alleles; *AR*, allelic richness; *H*_*O*_, average observed heterozygosity; *H*_*E*_, average expected heterozygosity; *F*_*IS*_, average inbreeding coefficient; *n*_*H*_, number of haplotypes (cyt *b*); *n*_*HP*_, number of private haplotypes (cyt *b*); values are statistical significant.

### Morphology

**Fig 4:**
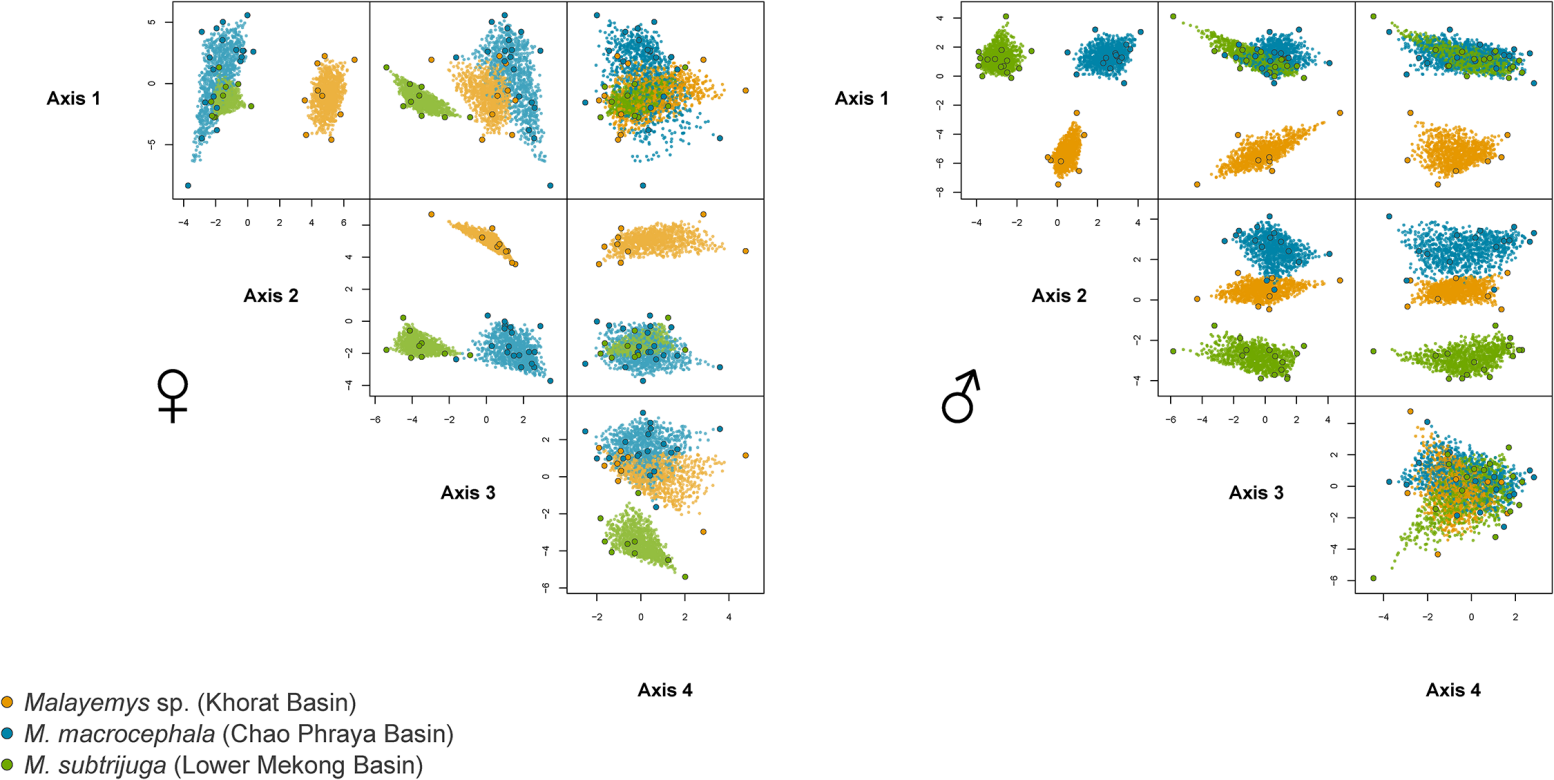
Morphological differences in shell characteristics as well as coloration pattern of *Malayemys macrocephala*, *M*. *subtrijuga* and the *Malayemys* from the Khorat Basin. Four-dimensional hypervolumes based on minimum convex polytopes of morphological variables for each sex are shown to the right. Axes refer to the first four principal components.

The four-dimensional hypervolumes of the three genetically delineated groups showed no overlap at all (Soerensen overlap = 0.0 between all pairs), neither in males nor in females (Fig 4). Complete differentiation in morphological space is observed among the genetically defined clades (Table 3). For variable contribution to the respective principal components, see S1 Text, for properties of the hypervolumes, see S4 Table.

**Table 3:** Morphological characters for studied individuals of *M*. *macrocephala*, *M*. *subtrijuga* and *Malayemys* from the Khorat Basin.

Character	*M*. *macrocephala*	*M*. *macrocephala*	*M*. *subtrijuga*	*M*. *subtrijuga*	*Malayemys* (Khorat)	*Malayemys* (Khorat)
	♂ (*n* = 25)	♀ (*n* = 26)	♂ (*n* = 15)	♀ (*n* = 10)	♂ (*n* = 9)	♀ (*n* = 9)
SCL*	108.8 ± 17.59 (74–156)	139.69 ± 41.37 (78–220)	100.13 ± 15.74 (76–132)	94.6 ± 10.21 (83–115)	108.56 ± 23.6104 (87–155)	115.11 ± 41.02 (82–191)
SPL*	86.6 ± 12.8 (61–117)	117.38 ± 34.29 (63–176)	82 ± 12.72 (63–107)	79.1 ± 9.31 (68–100)	88.89 ± 16.7 (74–122)	93.22 ± 31.42 (67–163)
CCL*	120.96 ± 20.37 (80–178)	153.54 ± 42.29 (86–225)	111.67 ± 16.01 (86–145)	106 ± 11.49 (92–128)	122.33 ± 26.24 (98–176)	131.33 ± 46.45 (94–210)
SCW*	79.92 ± 10.65 (60–102)	108.46 ± 29.73 (63–158)	75.27 ± 10.12 (59–96)	73.9 ± 6.87 (66–88)	83.22 ± 14.92 (71–114)	87.67 ± 25.05 (67–143)
CCW*	101 ± 13.77 (74–129)	139.12 ± 38.22 (80–202)	99.53 ± 12.13 (78–122)	97.6 ± 9.34 (84–111)	106.67 ± 15.13 (90–136)	121.67 ± 40.91 (89–194)
HT*	44.28 ± 5.53 (34–59)	59.15 ± 16.11 (35–90)	43.4 ± 4.53 (36–53)	43 ± 3.62 (37–49)	45.11 ± 7.44 (37–61)	47.78 ± 11.1 (39–75)
NL*	10.64 ± 1.8 (7–15)	12 ± 3.14 (6–17)	8.67 ± 1.35 (6–11)	8.4 ± 1.17 (6–10)	10.89 ± 3.66 (7–19)	11.44 ± 4.36 (8–20)
NW*	7.04 ± 2.17 (2–11)	8.69 ± 3.7 (3–16)	8.07 ± 2.09 (5–13)	9 ± 2.4 (5–13)	10.22 ± 1.3 (8–12)	11.00 ± 3.54 (5–16)
V1L*	21.36 ± 3.26 (15–29)	27.96 ± 8.94 (7–43)	20.53 ± 3.31 (17–28)	19.1 ± 3.41 (12–25)	21.89 ± 4.88 (16–30)	23.22 ± 7.51 (15–37)
V1W*	14.76 ± 3.15 (8–22)	20.23 ± 7.19 (10–41)	13.4 ± 2.03 (10–17)	13.1 ± 1.37 (11–15)	18.11 ± 5.49 (12–30)	19.78 ± 6.51 (12–32)
V2L*	18.36 ± 3.29 (13–28)	25.08 ± 7.53 (14–40)	17.47 ± 2.47 (14–22)	18.1 ± 3.41 (15–27)	18.44 ± 3.84 (15–28)	21.78 ± 7.28 (15–34)
V2W*	21.21 ± 3.31 (15–29)	30.2 ± 9.68 (14–50)	19.53 ± 2.59 (16–24)	19.1 ± 2.18 (16–22)	23.44 ± 3.64 (18–30)	25.44 ± 7.99 (17–41)
V3L*	18.4 ± 3.88 (11–29)	24.77 ± 8.1 (12–46)	16.67 ± 4.29 (11–29)	17 ± 2.87 (14–22)	19.67 ± 4.39 (16–30)	21.44 ± 7.75 (15–35)
V3W*	22.12 ± 3.57 (15–31)	31.12 ± 10.06 (16–53)	19.8 ± 2.96 (14–25)	18.89 ± 2.03 (16–22)	24.56 ± 4.95 (18–34)	26.89 ± 8.37 (17–44)
V4L*	17.44 ± 3.18 (11–27)	22.35 ± 5.89 (13–32)	16.27 ± 2.43 (11–20)	14.11 ± 1.83 (10–17)	18.13 ± 4.12 (14–27)	19.22 ± 6.57 (14–31)
V4W*	25.32 ± 4.64 (16–38)	33.5 ± 10.97 (17–56)	22.93 ± 4.23 (15–31)	20.89 ± 2.03 (18–25)	26.75 ± 7.48 (19–42)	28.44 ± 11.35 (19–51)
V5L*	22.96 ± 4.91 (15–37)	26.38 ± 8.12 (10–39)	20.73 ± 4.65 (11–30)	19.4 ± 2.22 (17–23)	23.63 ± 6.5 (12–34)	22.44 ± 8.13 (16–37)
V5W*	28.28 ± 4.56 (19–38)	37.58 ± 10.64 (18–58)	27.07 ± 5.6 (16–39)	25.2 ± 2.25 (22–29)	32.13 ± 7.72 (24–46)	33.00 ± 12.6 (22–57)
CPL*	87.92 ± 12.95 (62–120)	121.12 ± 35.78 (66–182)	83.33 ± 12.84 (63–108)	80.5 ± 8.93 (69–100)	90.78 ± 16.75 (75–123)	98.56 ± 36.89 (69–171)
SPW*	68.96 ± 11.87 (51–110)	93.27 ± 26.23 (53–142)	63.6 ± 8.42 (50–81)	62.1 ± 6.03 (54–74)	66.22 ± 9.73 (56–84)	70.44 ± 19.31 (53–116)
CPW*	77.2 ± 8.64 (58–97)	104 ± 28.01 (59–155)	71.27 ± 9.37 (56–93)	69.7 ± 6.88 (61–85)	71.56 ± 10.48 (61–90)	79.33 ± 21.74 (58–121)
GulL*	12.08 ± 2.41 (9–18)	14.62 ± 4.56 (7–23)	11.33 ± 2.13 (7–17)	11.2 ± 1.93 (9–16)	13.44 ± 4.1 (9–22)	14.56 ± 6.42 (9–28)
HumL*	11.88 ± 2.35 (7–15)	16.73 ± 5.2 (8–25)	10.27 ± 2.15 (6–15)	10.5 ± 1.51 (9–13)	11.00 ± 2.0 (8–14)	11.44 ± 4.69 (8–20)
PecL*	11.12 ± 1.96 (7–15)	16.04 ± 5.03 (8–26)	13.8 ± 2.31 (10–17)	13.1 ± 1.45 (11–16)	14.78 ± 2.54 (12–20)	16.11 ± 6.33 (10–28)
AbdL*	22 ± 3.45 (13–30)	31.77 ± 10.35 (16–51)	20.07 ± 3.61 (14–28)	18.7 ± 2.83 (15–25)	21.78 ± 6.04 (16–32)	23.56 ± 8.34 (17–39)
FemL*	16.88 ± 3.63 (12–27)	20.81 ± 5.33 (12–30)	14.47 ± 3.04 (10–21)	14.1 ± 2.42 (10–18)	17.56 ± 2.46 (14–22)	18.56 ± 8.88 (12–29)
AnL*	13.28 ± 2.59 (8–19)	18.62 ± 5.85 (10–30)	12.2 ± 1.82 (10–16)	11.8 ± 1.32 (10–15)	12.00 ± 2.12 (9–16)	13.44 ± 5.03 (10–23)
AfW*	18.96 ± 4.2 (12–30)	20.52 ± 6.12 (11–34)	14.87 ± 2.33 (12–20)	14.4 ± 2.01 (12–19)	16.56 ± 3.84 (13–24)	16.00 ± 5.24 (10–26)
Ppigm	33.74 ± 11.58 (10–56)	33.54 ± 9.72 (16–50)	38.53 ± 11.16 (17–58)	37 ± 6.75 (25–47)	52.89 ± 14.62 (36–78)	44.44 ± 10.70 (35–65)
NasS	3.2 ± 0.96 (2–4)	3.08 ± 0.98 (2–4)	6.53 ± 1.3 (5–10)	6.5 ± 2.27 (4–11)	2.78 ± 0.97 (2–4)	2 ± 0 (2–2)
OcuR	1.5 ± 0.51 (1–2)	1.52 ± 0.51 (1–2)	2 ± 0 (2–2)	2 ± 0 (2–2)	1 ± 0 (1–1)	1 ± 0 (1–1)
OcuRCharc	0% pronounced, 40% distinct, 44% medium, 4% weak	3.8% pronounced, 30.8% distinct, 38.5% medium, 7.7% weak	100% pronounced, 0% distinct, 0% medium, 0% weak	100% pronounced, 0% distinct, 0% medium, 0% weak	11.1% pronounced, 0% distinct, 22% medium, 55.5% weak	22% pronounced, 0% distinct, 22% medium, 55.5% weak
NuchSh	28% broad, 72% narrow	30.8% broad, 69.2% narrow	66.7% broad, 33.37% narrow	80% broad, 20% narrow	100% broad, 0% narrow	88.8% broad, 0% narrow
MargCol	0% blotches, 72% bars, 28% black	3.8% blotches, 96.2% bars, 0% black	0% blotches, 93.3% bars, 6.7% black	0% blotches, 100% bars, 0% black	78% blotches, 0% bars, 22% black	100% blotches, 0% bars, 0% black
ChinS	8% present, 92% absent	11.6% present, 88.5% absent	66.7% present, 33.3% absent	80% present, 20% absent	0% present, 100% absent	11% present, 89% absent
EyeCol	0% brown, 68% white, 20% green	0% brown, 53.8% white, 26.9% green	0% brown, 100% white, 0% green	0% brown, 100% white, 0% green	44% brown, 44% white, 0% green	55% brown, 44% white, 0% green
InfLorbCon	8% present, 92% absent	3.8% present, 96.2% absent	80% present, 20% absent	80% present, 20% absent	0% present, 100% absent	0% present, 100% absent
InfLorbL	100% present, 0% absent	100% present, 0% absent	100% present, 0% absent	100% present, 0% absent	33% present, 67% absent	0% present, 100% absent
InfLorb	16% narrow, 84% broad	19.2% narrow, 80.8% broad	100% narrow, 0% broad	100% narrow, 0% broad	88.8% narrow, 11.1% broad	100% narrow, 0% broad
InfLorbSh	0% angulate, 88% curved, 12% straight	0% angulate, 96.2% curved, 3.8% straight	100% angulate, 0% curved, 0% straight	100% angulate, 0% curved, 0% straight	0% angulate, 22% curved, 78% straight	0% angulate, 11% curved, 89% straight
postOcS	96% present, 4% absent	100% present, 0% absent	46.7% present, 53.3% absent	60% present, 40% absent	0% present, 100% absent	11% present, 89% absent

* metric characteristics: mean values, standard deviation, maximum and minimum values (given in brackets) given in mm.

SCL: straight carapace length; SPL: straight plastron length; CCL: curved carapace length; SCW: straight carapace width; CCW: curved carapace width; HT: shell height; NL: length of nuchal scute; NW: width of nuchal scute; V1L, V2L, V3L, V4L, V5L: length of vertebral scutes 1–5; V1W, V2W, V3W, V4W, V5W: width of vertebral scutes 1–5; CPL: curved plastron length; SPW: straight plastron width; CPW: curved plastron width; GulL, HumL, PecL, AbdL, FemL, AnL: medial seam length of plastral scutes; AfW: width of anal fork; Ppigm: pigmentation of plastron in %; NasS: number of nasal stripes; OcuR: number of ocular rings; OcuRCharc: feature characteristics of ocular rings; NuchSh: shape of nuchal scute; MargCol: coloration patterns of lower marginal scutes; ChinS: presence of chin stripe; EyeCol: eye coloration; InfLorbCon: presence of connection of infraorbital stripe with crown; InfLorbL: length of infraorbital stripe; InfLorb: connection of infraorbital stripe to loreal seam broad or narrow; InfLorbSh: shape of infraorbital stripe; postOcS: presence of postocular stripe.

### Species distribution model

The predictive model performed well with high area under the receiver operating characteristic curve values (AUC_training_ = 0.86, AUC_test_ = 0.85), indicating the model to discriminate well between suitable and unsuitable environmental space. Mean temperature of the warmest quarter (bio 10) was the variable with the highest importance across all 100 replicates (44.3%), followed by annual mean temperature (bio 1, 39.9%) and precipitation of the driest quarter (bio 17, 5.12%). All other variables contributed less than 5% (S6 Table).

**Fig 5:**
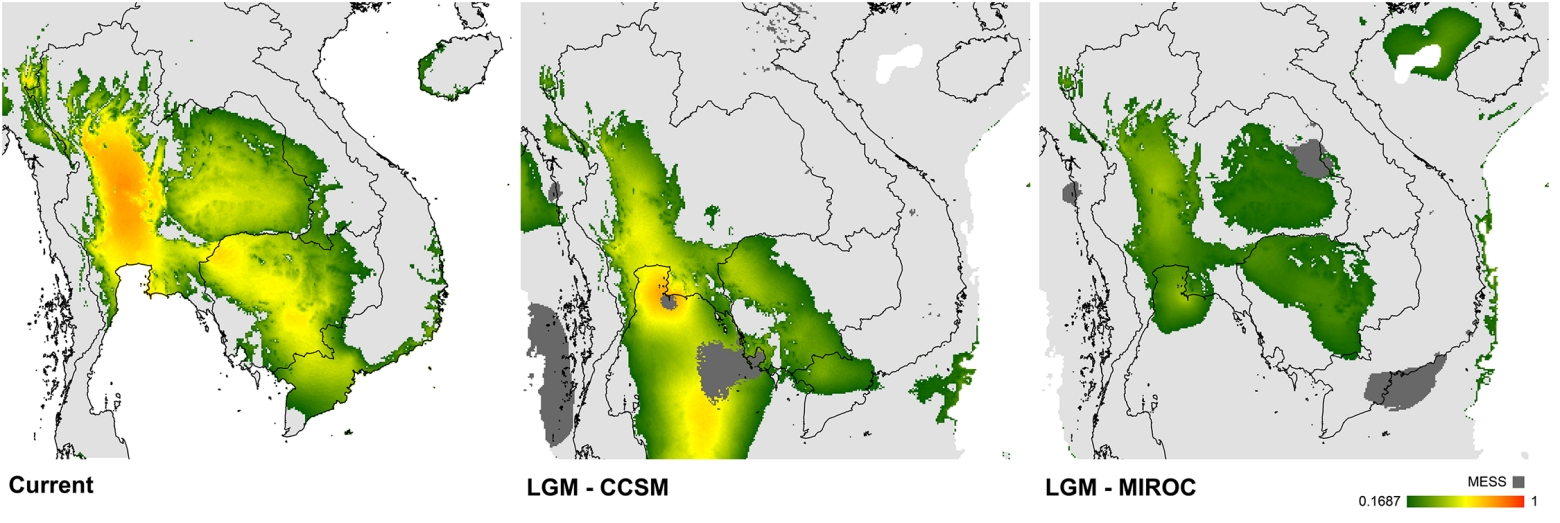
Potential distribution of suitable climate for *Malayemys*. **left**: present distribution of suitable climate as derived from a maximum entropy model; **middle**: projection onto paleoclimatic conditions of the Last Glacial Maximum (21 Ka) derived from the global circulation model CCSM; **right**: projection onto paleoclimatic conditions of the Last Glacial Maximum derived from the global circulation model MIROC. Suitability ranging from moderate (green) to high (red). Extrapolation area is displayed in dark grey.

Under present climatic conditions, the model reflects the known distribution range of *Malayemys*. Areas of higher altitude such as the Dangrek Mountain range (separating the Khorat Basin from the Northern Plains of Cambodia) and the Cardamom Mountains (separating eastern Thailand from the south-west of Cambodia) are not predicted to have suitable environmental conditions (Fig 5). Projections onto paleoclimatic conditions derived from two different global circulation models, however, suggest that suitable environmental space contracted to largely disjunct ranges during the last LGM. The first of these areas, corresponding to the current distribution range of *M*. *macrocephala*, was predominantly confined to the Chao Phraya Basin, but extended southwards onto the Sunda Shelf. A second suitable area, which was perhaps intermittently connected to the first via a narrow corridor exhibits lower occurrence probabilities and stretched from eastern Thailand across the Lower Mekong Basin to the Mekong Delta in southern Vietnam. This area matches the current distribution range of *M*. *subtrijuga*. Only the MIROC projection predicts extensive suitable environmental conditions within the Khorat Basin during the LGM, wherein the potential distribution in this region was much more restricted in CCSM but still present.

## Taxonomic Conclusions

Our analyses revealed substantial phylogeographic structure within the genus *Malayemys*, with three mitochondrial clades that largely correspond to genetic clusters identified by structure analyses of 12 microsatellite loci and to drainage basins. Two of these entities, from the Chao Phraya Basin and from the Lower Mekong Basin, can be identified with the species *Malayemys macrocephala* (Gray, 1859) and *M*. *subtrijuga* (Schlegel & Müller, 1845), respectively. Based on morphological differences alone, the validity of these two taxa have been recognized since the studies of Brophy [1,6]. According to our data, mitochondrial sequence divergence is relatively low between *M*. *macrocephala* and *M*. *subtrijuga* (Fig 2), with uncorrected *p*-distances of 1.45% for the DNA fragments comprising the cyt *b* gene and 1.09% for the one comprising the partial ND4 gene (S1 Fig, S1 FASTA). Despite this weak differentiation, our microsatellite analyses revealed distinct nuclear genomic gene pools, suggesting reproductive isolation and corroborating their species status. However, mismatches of mitochondrial haplotypes and structure clusters (Figs 2 and 3) indicate old mitochondrial introgression into *M*. *macrocephala*.

The populations from the Khorat Basin constitute another morphologically distinct group (Fig 4), harbouring the most divergent mitochondrial lineage (see also S1 Fig). As in *M*. *macrocephala* and *M*. *subtrijuga*, microsatellite data indicate its reproductive isolation. Based on the congruence of multiple lines of evidence, we conclude that the Khorat populations represent a distinct third species for which no name is available (cf. [70,71]). This species will be formally described below.

***Malayemys khoratensis* sp. nov.** urn:lsid:zoobank.org:act:B88DB370-C3D0-4E64-A79A-3A77DDE7BCD8

**Fig 6:**
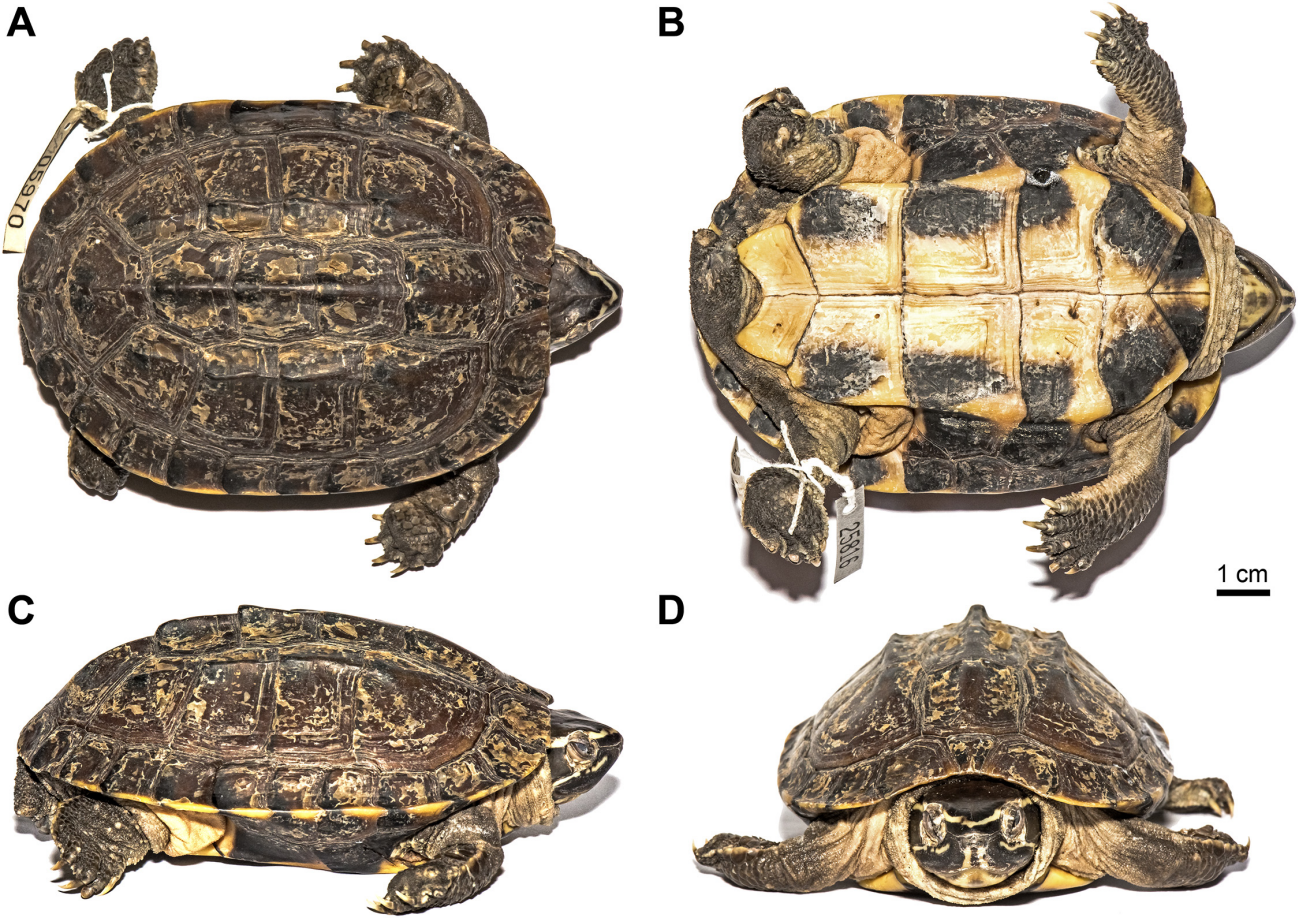
(A) Dorsal, (B) ventral, (C) lateral, and (D) frontal views of the holotype of *Malayemys khoratensis* (THNHM 25816, young, adult female from Udon Thani, Thailand).

Holotype: THNHM 25816, young, adult female (Fig 6) from Udon Thani, Udon Thani Province, Thailand (17.36555°N, 102.81427°E, WGS 1984), collected in July 2014 by FI and MC.

**Fig 7:**
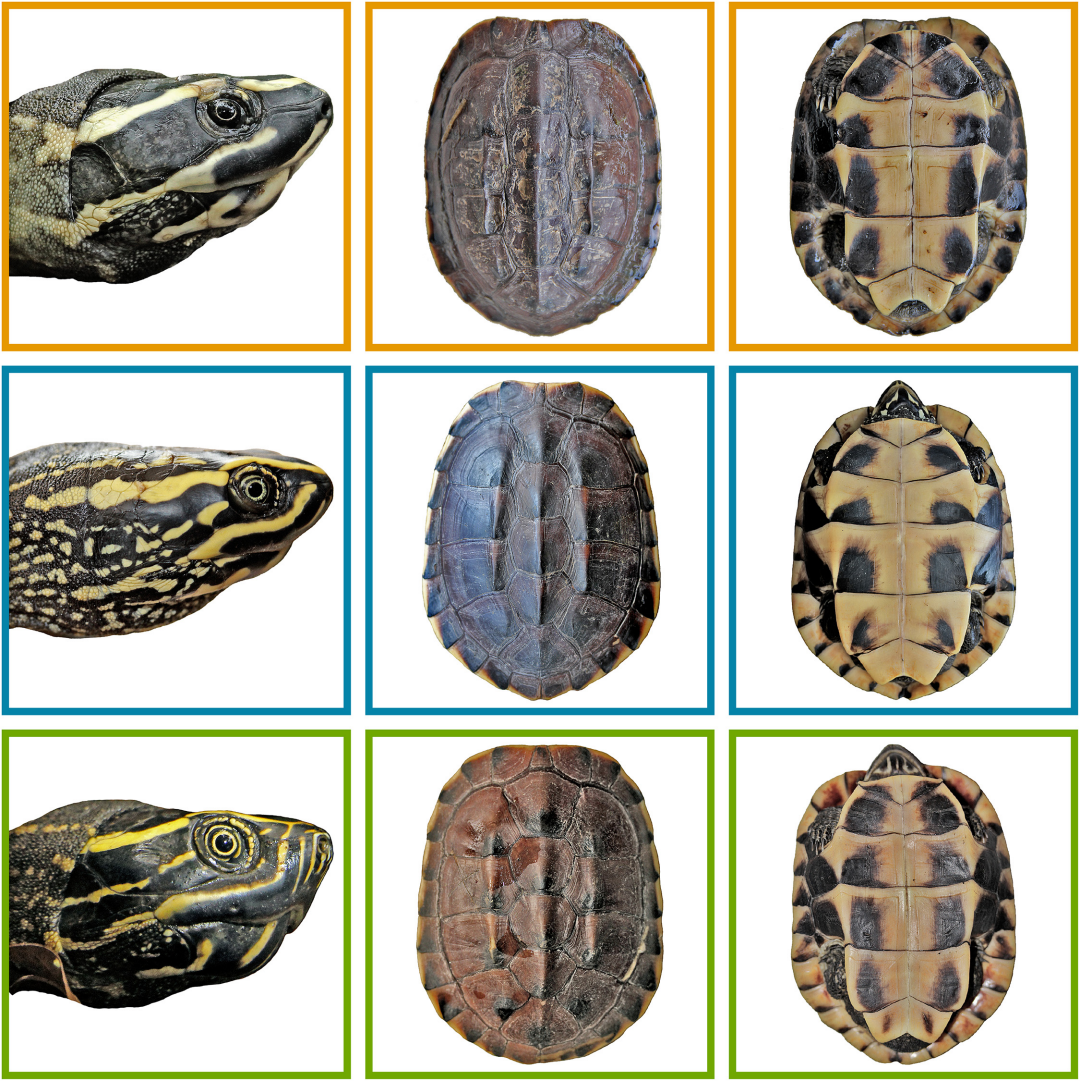
Morphological differences in shell characteristics and head colouration patterns of *Malayemys khoratensis* (orange), *Malayemys macrocephala* (blue), *Malayemys subtrijuga* (green).

**Diagnosis**: *Malayemys* species with a straight carapace length below 20 cm and a tricarinate, blackish-brown carapace, and a blackish-brown head with distinct yellowish facial stripes. *Malayemys khoratensis* differs from *M*. *macrocephala* by the following characters: (1) first vertebral scute roughly square and not tapered posteriorly (V1W/V1L: 0.83±0.09 vs. 0.74±0.19 in females, 0.83±0.12 vs. 0.69±0.09 in males); (2) lower marginal scutes 8–12 with a pattern of diagonal to cone-shaped blackish brown blotches originating from the outer posterior corner vs. narrow blackish-brown bars at the posterior sutures; (3) infraorbital stripe not or rarely reaching the loreal seam, not broadened at the suture vs. always reaching the loreal seam and usually distinctly broadened at the suture; (4) infraorbital stripes only slightly curved below anterior edge of eyes vs. distinctly curved or angled; (5) short yellowish postocular stripe (between supraorbital and infraorbital stripe) lacking or reduced vs. postocular stripe always present and distinct (Fig 7).

*Malayemys khoratensis* differs from *M*. *subtrijuga* by the following characters: (1) first vertebral scute roughly square not tapered posteriorly (V1B/V1L: 0.83±0.09 vs. 0.7±0.11 in females, 0.83±0.12 vs. 0.66±0.8 in males); (2) lower marginal scutes 8–12 with a pattern of distinct diagonal to cone like blackish brown blotches originating from the outer posterior corner vs. narrow blackish bars; (3) infraorbital stripe not or rarely reaching the loreal seam vs. extending across loreal seam and often joining the supraorbital stripe; (4) two yellowish nasal stripes below nostrils vs. four or more nasal stripes; (5) infraorbital stripes only slightly curved below eyes vs. distinctly angled below anterior edge of eyes; (6) a single yellowish orbital ring vs. two well recognizable yellowish orbital rings (Fig 7).

Genetically, *M*. *khoratensis* differs from both congeners by the presence of tyrosine (T) instead of cytosine (C) at positions 45, 48, 117, 144 and by the presence of cytosine (C) instead of tyrosine (T) at positions 111, 387, 388,615, 616 of the 1078-bp-long reference alignment of the cyt *b* gene (S1 FASTA).

**Description of the holotype** (Fig 6): Straight carapace length 106 mm; carapace moderately domed, somewhat depressed; carapace with a prominent dorsal keel from first to fifth vertebral and two nearly parallel lateral keels from the first to forth costal, most prominent on the second and third costal, feeble on forth costal; nuchal scute rather triangular, nearly as broad as long and not projecting beyond front border of adjacent marginals; first vertebral almost squarish (22x18 mm), anterior of scute slightly convex; second vertebral almost square (18x21 mm); third vertebral faintly hexangular and slightly broader than long (17x22 mm); forth vertebral hexangular (17x23 mm); fifth vertebral trapezoid with a convex posterior scute (25x33 mm); first costal quadrant, second and third costal broader than long, forth costal obliquely quadrangular; plastron shorter than carapace; slightly rounded anteriorly, posterior edges of gulars form an angle of 110°; a broad bridge, plastral edge slightly angular; measurements of median sutures of plastral scutes: gular 12 mm; humeral 9 mm, pectoral 14 mm, abdominal 22 mm, femoral 20 mm, anal 10 mm.

Toes fully webbed, claws moderate; posterior region of thighs and area above tail with a few very small conical and pointed scales; two rows of flat scales under the tail.

Head large, skin of top smooth with a large undivided prefrontal scute. Supraocular scute very large, bordering the upper jaw scute. Upper jaw notched.

Carapace dark brown; marginal scutes exhibit blackish area along the posterior edge; submarginal scutes: 1st almost entirely yellowish, 2nd and 3rd with blackish area near sutures; 4th to 7th (next to bridge) almost entirely blackish, 8th to 12th (posterior to bridge) triangular to cone-like blackish area emanating from outer posterior corner of scutes; plastron pale yellow, each scute except anal scutes with a large blackish area emanating from the lateral posterior corner; head blackish brown; one ivory-white supraorbital stripe (pale yellowish in life) running from tip of snout above eye and ear to base of neck; one ivory infraorbital stripe (pale yellowish in life) from about anterior lower corner of eye, crossing jaw angle, rather straight running continuously below ear to neck; two parallel ivory lines (pale yellowish in life) from nostrils to upper jaw, more or less continuous with two pale washed-out median lines crossing lower jaw; soft parts of posterior neck and shoulder greyish beige; limbs darker on upper surfaces.

**Paratypes**: THNHM 25999, adult female, Udon Thani, Thailand); ZFMK 97198, adult male, Udon Thani, Thailand; MTD 49150, female, Udon Thani, Thailand.

**Variation**: For variation in colour traits and morphological measurements, see Table 3. For genetic variation, see GenBank accession numbers (Table 1).

**Etymology**: The species epithet refers to the Khorat Basin, the watershed to which the range of the new species appears to be restricted. The proposed English common name is Khorat snail-eating turtle.

**Distribution**: *M*. *khoratensis* is so far only known from the Khorat Basin of northeastern Thailand. More precisely from Sikhio District, Nakhon Ratchasima Province, a location drained by the Mun River, and the provinces of Udon Thani (type locality) and Nong Bua Lamphu, in areas associated with the Chi River, a tributary of the Mun River.

## Discussion

Different data sets (morphology, mtDNA, and microsatellite loci) support our hypothesis that *Malayemys* consists of three clearly distinct taxa. Quantitative analyses yielded a complete morphological differentiation of all three clusters. This lack of overlap in morphological space is a strong argument for specific distinctiveness, as one would not expect such discrete clusters if there was consistent exchange between sampled populations. Furthermore, all three species can be identified by qualitative, diagnostic characters (see above). While *M*. *macrocephala* and *M*. *subtrijuga* have already been granted species status based on morphology [5,6], the third species described herein is new to science. At least for the new species *M*. *khoratensis*, mitochondrial and microsatellite markers showed largely congruent patterns, both supporting its species status. According to our phylogeny, the initial—comparatively deep—split within *Malayemys* separated *M*. *khoratensis* from the last common ancestor of its two congeners. Observed *p*-distances between *M*. *khoratensis* and the other two species resemble those of many well-established turtle species (e.g. [17,18]), whereas divergences between *M*. *macrocephala* and *M*. *subtrijuga* are close to the lowermost divergence values observed between distinct turtle species [72,73]. However, population genetic analyses revealed no signs of gene flow between any of the three *Malayemys* species (no admixture among structure clusters; high F_*ST*_ values and a high number of private alleles and haplotypes; see e.g. [74]) and thus corroborate to treat all three taxa as full species under the restrictive biological species concept [75,76]. However, considering the shared mitochondrial haplotypes in *M*. *macrocephala* and *M*. *subtrijuga*, some historical introgression seems to have occurred between these two species.

Although *Malayemys* is excessively traded and used for religious release, our study found well-separated distribution ranges and identified only a few genetically mismatching individuals that may result from translocation by humans. This could indicate that turtles used for traditional religious release are mostly locally caught and released locally. Yet, for three mismatching turtles translocation seems likely: a single individual from within the range of *M*. *subtrijuga*, offered for religious release in Siem Reap, Cambodia (Site 24; Lower Mekong Basin), was morphologically and genetically allocated to *M*. *macrocephala*, suggestive of introduction from Thailand. Another two individuals from the Khorat Plateau (site 18; Sakhon Nakhon) genetically as well as morphologically represented *M*. *macrocephala* and not, as expected, *M*. *khoratensis*. In addition, a single *M*. *macrocephala* has been reported from the Nam Lik Valley, Vientiane Province, northern Laos [77] and *M*. *macrocephala* was found on food markets in and around Vientiane city [78]. It remains unclear whether these turtles have been introduced or whether *M*. *macrocephala* occurs naturally in the region of the northern Middle Mekong and its tributaries.

Snail-eating turtles occur in a variety of natural and anthropogenic freshwater habitats, including rice fields and canals across the Southeast Asian lowlands but are absent from higher elevations [1,6]. In accordance, our SDM identifies temperature-related variables as the limiting parameters for geographic distribution, restricting the occurrence of *Malayemys* to lower elevations. The present distribution pattern has to be understood as the result of paleogeographic events, historical climate fluctuations and eustatic sea level changes.

During the last glacial maximum, the sea level fell and exposed a vast portion of the Sunda Shelf as swampy plains that might have served as suitable habitat for the semi-aquatic *Malayemys*. However, rising sea levels led to inundation of these potentially suitable areas during the Pleistocene and post-Pleistocene period, making vast areas of the Asian continental shelf uninhabitable for *Malayemys* once more.

In addition, tectonic activity caused significant rearrangement of the Southeast Asian drainage systems leading to alterations of evolutionary and ecological trajectories [79–82]. Uplifting along the southern and western edges of the Khorat Plateau disconnected the Mun River, nowadays a tributary of the Mekong, from the Chao Phraya Basin [83–86]. The predictive SDM under current climatic conditions highlights geographic barriers (i.e., mountain ranges) that separate suitable areas in the Khorat Basin from the remaining areas suitable for *Malayemys*. Past projections of the SDM suggest that environmentally suitable space for *Malayemys* was restricted to two disjunct areas during the LGM. These two potentially suitable glacial distribution ranges largely match the current ranges of *M*. *macrocephala* and *M*. *subtrijuga*, proposing that the divergence of these two species occurred in allopatry and was triggered by Pleistocene climate fluctuations. Yet, the older split, separating *M*. *khoratensis*, is difficult to explain. Theoretically, it should be assumed that for this species a third distinct glacial distribution range existed. However, the LGM model revealed no suitable areas within the current range of the new species, which suggests either a niche shift (i.e., the realized niche of *M*. *khoratensis* has changed through time) or that *M*. *khoratensis* could have co-existed during the LGM with *M*. *macrocephala* and *M*. *subtrijuga* in the same areas.

The presence of Pleistocene (or older) fossils belonging to the freshwater turtle genera *Chitra* and *Batagur* as well as sediments obtained from Khok Sung, Nakhon Ratchasima Province, Thailand suggest that the Mun River was historically draining into the Chao Phraya instead of flowing from west to east into the Mekong system [85]. Now extirpated from east of the Chao Phraya, this historical drainage system arrangement likely facilitated gene flow between western and southern *Batagur* populations such as the *B*. *trivittata* and its closest relative *B*. *borneoensis* [85]. Therefore, the Khorat Basin might have served as a historical migration route for freshwater turtles between the Lower Mekong and the Chao Phraya Basin. These hydrogeographical events influenced geographic distribution patterns and genetic diversity of freshwater fishes (*Henicorhynchus*), gharials (*Gavialis*), and semi-aquatic mud snakes (*Enhydris*) [85–88].

## Supporting Information

S1 FASTA Reference alignment of the nearly complete mitochondrial cytochrome *b* gene (plus adjacent DNA coding for tRNA-Thr) of *Malayemys*. (FAS)

S1 Fig Unrooted haplotype networks constructed under statistical parsimony for cyt *b* and ND4 sequences showing relationships among and between haplotypes of *Malayemys*. Circle size is relative to haplotype frequency, dots represent extinct or unsampled haplotypes. Coloration corresponds to clusters identified by the microsatellite analyses.
(PDF)

S1 Table PCR conditions for mtDNA fragments. (DOCX)

S2 Table Microsatellite loci, allele size ranges and number of alleles of the individual loci. For primer sequences, see original references. Forward primers were fluorescent-labelled.
(DOCX)

S3 Table Pairwise fixation indices (*F*_ST_ values) for the structure clusters (*K* = 3) of *Malayemys*. All *F*_ST_ values are significantly different from zero.
(DOCX)

S4 Table Volumes of four dimensional hypervolumes. (DOCX)

S5 Table Average uncorrected *p*-distances of cyt *b* and ND4 within *Malayemys*. (DOCX)

S6 Table Bioclimatic variables used for species distribution models and variable contributions. (DOCX)

S1 Text Detailed description of examined morphological characters and contribution of variables to PCA. (DOCX)
