# Supplementary figures and images for: Integrative Taxonomy of Southeast Asian Snail-Eating Turtles (Geoemydidae: Malayemys) Reveals a New Species and Mitochondrial Introgression
Source: PLoS One. 2016 Apr 6;11(4):e0153108. doi: 10.1371/journal.pone.0153108 (PMC4822821; doi:10.1371/journal.pone.0153108)

**cyt *b***

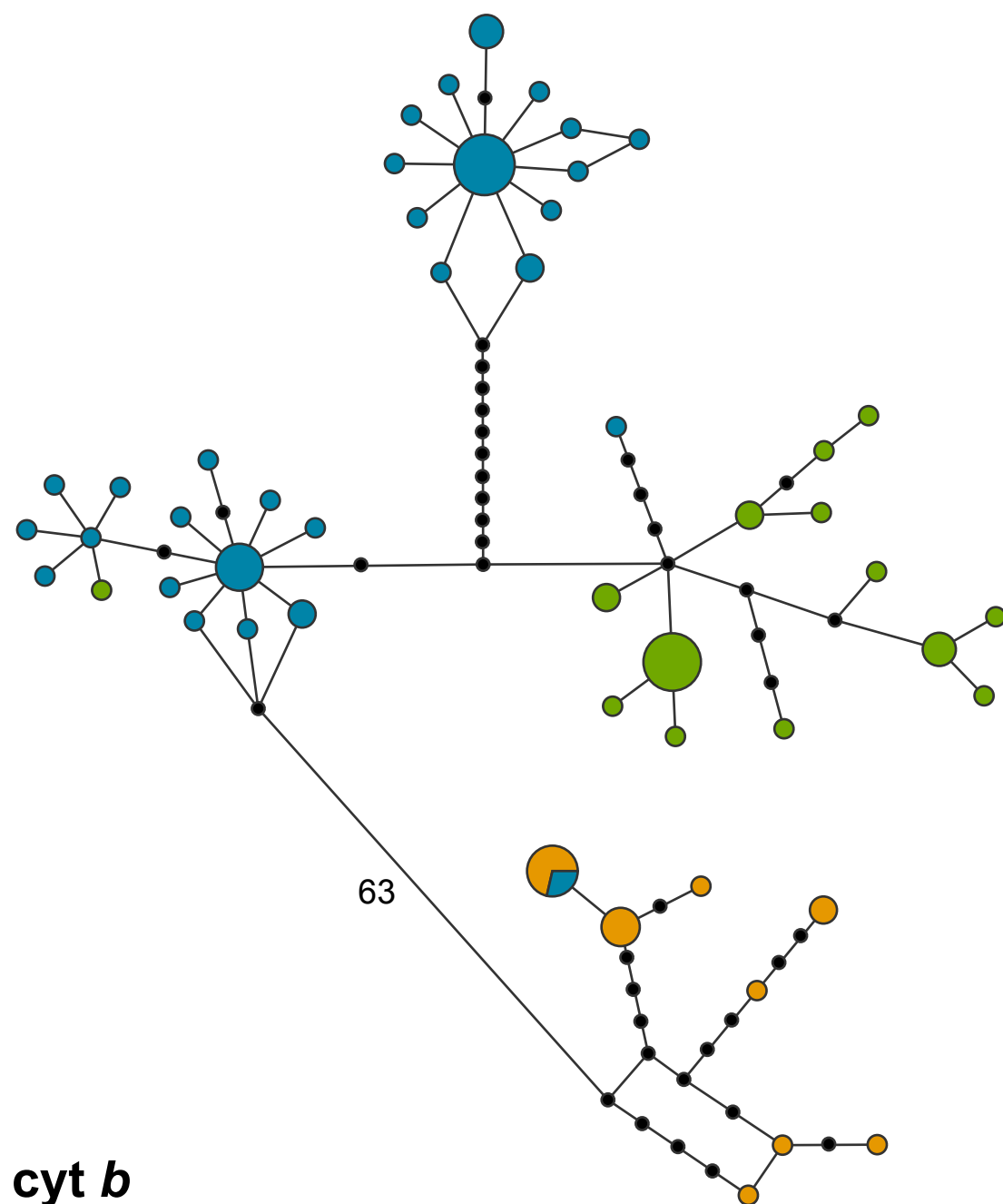

**ND4**

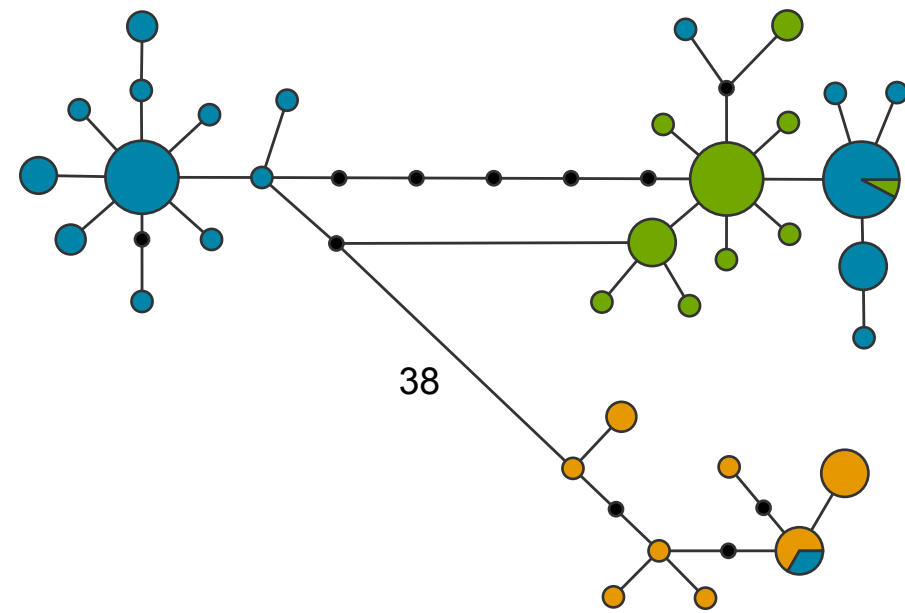

10  
①

● *M. macrocephala*

● *M. subtrijuga*

● *M. sp. nov.*

Supplement: S1 Fig — Circle size is relative to haplotype frequency, dots represent extinct or unsampled haplotypes. Coloration corresponds to clusters identified by the microsatellite analyses. (PDF) [file pone.0153108.s002.pdf]
