# Supplementary material for: Integrative Taxonomy of Southeast Asian Snail-Eating Turtles (Geoemydidae: Malayemys) Reveals a New Species and Mitochondrial Introgression
Source: PLoS One. 2016 Apr 6;11(4):e0153108. doi: 10.1371/journal.pone.0153108 (PMC4822821; doi:10.1371/journal.pone.0153108)
Supplement: S1 Table — (DOCX) [file pone.0153108.s003.docx]

Ihlow *et al.* Integrative Taxonomy of Southeast Asian Snail-eating Turtles (Geoemydidae: *Malayemys*) unravels a new species and mitochondrial introgression

**Supporting Information S1.** PCR conditions for mtDNA fragments.

|  | **Thermocycling conditions** | | | | | |
| --- | --- | --- | --- | --- | --- | --- |
| **DNA fragment** | **ID** | **C** | **D** | **A** | **E** | **FE** |
| Cyt *b* | 95°C, 5 min | 35 | 95°C, 45 s | 56°C, 30 s | 72°C, 60 s | 72°C, 8 min |
| ND4 | 94°C, 5 min | 40 | 94°C, 45 s | 53°C, 30 s | 72°C, 60 s | 72°C, 10 min |
| Abbreviations: ID = initial denaturation, C = number of cycles, D = denaturation, A = annealing, E = extension, FE = final extension. | | | | | | |
