# Supplementary material for: Integrative Taxonomy of Southeast Asian Snail-Eating Turtles (Geoemydidae: Malayemys) Reveals a New Species and Mitochondrial Introgression
Source: PLoS One. 2016 Apr 6;11(4):e0153108. doi: 10.1371/journal.pone.0153108 (PMC4822821; doi:10.1371/journal.pone.0153108)
Supplement: S2 Table — For primer sequences, see original references. Forward primers were fluorescent-labelled. (DOCX) [file pone.0153108.s004.docx]

Ihlow *et al.* Integrative Taxonomy of Southeast Asian Snail-eating Turtles (Geoemydidae: *Malayemys*) unravels a new species and mitochondrial introgression

**Supporting Information S2.** Microsatellite loci, allele size ranges and number of alleles of the individual loci. For primer sequences, see original references. Forward primers were fluorescent-labelled.

| **Locus** | **Original reference** | **Fluorescent label** | **Allele size range [bp]** | **Annealing T[°C]** | **Number of alleles** |
| --- | --- | --- | --- | --- | --- |
| Maucas01 | Vamberger *et al.* (2011) | ATTO 550 | 136-216 | 56 | 22 |
| Maucas06 | Vamberger *et al.* (2011) | ATTO 565 | 133-193 | 56 | 16 |
| Maucas18 | Vamberger *et al.* (2011) | 6-FAM | 222-294 | 56 | 18 |
| Test56 | Forlani *et al.* (2004) | HEX | 206-212 | 56 | 4 |
| Emys2 | Ciofi *et al.* (2009) | ATTO 550 | 167-205 | 56 | 20 |
| MR-3 | Mantziou *et al.* (2005) | ATTO 565 | 180-182 | 56 | 2 |
| GmuB08 | Roques *et al.* (2004) | ATTO 565 | 184-190 | 56 | 2 |
| Test10 | Forlani *et al.* (2004) | HEX | 180-210 | 56 | 12 |
| TWL221 | Perez *et al.* (2006) | ATTO 565 | 182-210 | 56 | 11 |
| TWI61 | Perez *et al.* (2006) | HEX | 243-265 | 56 | 11 |
| GP81 | Schwartz *et al.* (2003) | HEX | 359-383 | 56 | 6 |
| MR-5 | Mantziou *et al.* (2005) | HEX | 131-139 | 56 | 5 |
