# Supplementary material for: Integrative Taxonomy of Southeast Asian Snail-Eating Turtles (Geoemydidae: Malayemys) Reveals a New Species and Mitochondrial Introgression
Source: PLoS One. 2016 Apr 6;11(4):e0153108. doi: 10.1371/journal.pone.0153108 (PMC4822821; doi:10.1371/journal.pone.0153108)
Supplement: S3 Table — All FST values are significantly different from zero. (DOCX) [file pone.0153108.s005.docx]

Ihlow *et al.* Integrative Taxonomy of Southeast Asian Snail-eating Turtles (Geoemydidae: *Malayemys*) unravels a new species and mitochondrial introgression

**Supporting Information S4.** Pairwise fixation indices (*F_ST_* values) for the structure clusters (*K* = 3) of *Malayemys*. All *F_ST_* values are significantly different from zero.

|  | **Chao Phraya** | **Lower Mekong** | **Khorat** |
| --- | --- | --- | --- |
| **Chao Phraya** | - |  |  |
| **Lower Mekong** | 0.381 | - |  |
| **Khorat** | 0.438 | 0.553 | - |
