# Supplementary material for: Integrative Taxonomy of Southeast Asian Snail-Eating Turtles (Geoemydidae: Malayemys) Reveals a New Species and Mitochondrial Introgression
Source: PLoS One. 2016 Apr 6;11(4):e0153108. doi: 10.1371/journal.pone.0153108 (PMC4822821; doi:10.1371/journal.pone.0153108)
Supplement: S4 Table — (DOCX) [file pone.0153108.s006.docx]

Ihlow *et al.* Integrative Taxonomy of Southeast Asian Snail-eating Turtles (Geoemydidae: *Malayemys*) unravels a new species and mitochondrial introgression

**Supporting Information S5.** Volumes of four dimensional hypervolumes.

| Species | volume ♀ | n♀ | volume ♂ | n♂ |
| --- | --- | --- | --- | --- |
| *M. macrocephala* | 18.902 | 19 | 14.853 | 15 |
| *M.* sp. Nov. | 8.925 | 9 | 6.908 | 7 |
| *M. subtrijuga* | 8.984 | 9 | 14.205 | 15 |
