# Supplementary material for: Integrative Taxonomy of Southeast Asian Snail-Eating Turtles (Geoemydidae: Malayemys) Reveals a New Species and Mitochondrial Introgression
Source: PLoS One. 2016 Apr 6;11(4):e0153108. doi: 10.1371/journal.pone.0153108 (PMC4822821; doi:10.1371/journal.pone.0153108)
Supplement: S5 Table — (DOCX) [file pone.0153108.s007.docx]

Ihlow *et al.* Integrative Taxonomy of Southeast Asian Snail-eating Turtles (Geoemydidae: *Malayemys*) unravels a new species and mitochondrial introgression

**Supporting Information S6.** Average uncorrected *p*-distances of cyt *b* and ND4 within *Malayemys*.

| **Cyt *b*** |  |  |  |  |
| --- | --- | --- | --- | --- |
|  | *n* | *M. macrocephala* | *M. subtrijuga* | *M.* sp. nov |
| *M. macrocephala* | 25 | **0.14** |  |  |
| *M. subtrijuga* | 47 | 1.45 | **0.43** |  |
| *M.* sp. nov | 18 | 6.92 | 6.81 | **0.54** |
|  |  |  |  |  |
|  |  |  |  |  |
| **ND4** |  |  |  |  |
|  | *n* | *M. macrocephala* | *M. subtrijuga* | *M.* sp. nov |
| *M. macrocephala* | 25 | **0.17** |  |  |
| *M. subtrijuga* | 47 | 1.09 | **0.21** |  |
| *M.* sp. nov | 19 | 5.30 | 5.38 | **0.91** |

Values are percentages for a 1078 bp-long alignment of cyt *b* and a 785 bp-long alignment of the ND4 gene of *Malayemys* using the pairwise deletion option of MEGA 6.06 (Tamura et al. 2013). Between-group divergences are displayed below diagonal while within-group divergences are displayed on the diagonal in (bold), *n* is the number of sequences.
