# Supplementary material for: Integrative Taxonomy of Southeast Asian Snail-Eating Turtles (Geoemydidae: Malayemys) Reveals a New Species and Mitochondrial Introgression
Source: PLoS One. 2016 Apr 6;11(4):e0153108. doi: 10.1371/journal.pone.0153108 (PMC4822821; doi:10.1371/journal.pone.0153108)
Supplement: S6 Table — (DOCX) [file pone.0153108.s008.docx]

Ihlow *et al.* Integrative Taxonomy of Southeast Asian Snail-eating Turtles (Geoemydidae: *Malayemys*) unravels a new species and mitochondrial introgression

**Supporting Information S7.** Bioclimatic variables used for species distribution models and variable contributions.

| **Variable** | **Variable meaning** | **Variable contribution** |
| --- | --- | --- |
| bio1 | annual mean temperature | 39.88 |
| bio10 | mean temperature of warmest quarter | 44.25 |
| bio11 | mean temperature of coldest quarter | 1.62 |
| bio12 | annual mean precipitation | 4.76 |
| bio16 | precipitation of wettest quarter | 4.37 |
| bio17 | precipitation of driest quarter | 5.12 |
