# Supplementary material for: Integrative Taxonomy of Southeast Asian Snail-Eating Turtles (Geoemydidae: Malayemys) Reveals a New Species and Mitochondrial Introgression
Source: PLoS One. 2016 Apr 6;11(4):e0153108. doi: 10.1371/journal.pone.0153108 (PMC4822821; doi:10.1371/journal.pone.0153108)
Supplement: S1 Text — (DOCX) [file pone.0153108.s009.docx]

Ihlow *et al.* Integrative Taxonomy of Southeast Asian Snail-eating Turtles (Geoemydidae: *Malayemys*) unravels a new species and mitochondrial introgression

**Supporting Information S3.** Detailed description of examined morphological characters and contribution of variables to PCA.

**S3.1.** Examined morphological characters and abbreviations

**Metric characters:**

Straight carapace length (**SCL**): Straight-line distance from anterior to posterior carapace edge.

Straight carapace width (**SCW**): Straight-line distance at the level of the seam separating vertebral scutes 3 and 4.

Curved carapace length (**CCL**): Curved distance from anterior to posterior carapace edge.

Curved carapace width (**CCW**): Curved distance at the level of the seam separating vertebral scutes 3 and 4.

Nuchale length (**NL**): Length of Nuchal scute.

Nuchale width (**NW**): Width of posterior side of nuchal scute.

Vertebrale scute length (**V1L,** **V2L, V3L V4L, V5L**): Length of vertebral scute.

First vertebrale width (**V1W**): Maximum width of first vertebral scute measured at posterior edge.

Vertebrale width (**V2W, V3W, V4W, V5W**): Maximum width of vertebral scute measured across middle of scute.

Shell height (**HT**): Height of shell measured at the level of the seam separating vertebral scutes 3 and 4.

Straight plastron length (**SPL**): Straight-line distance from anterior to posterior plastron edge measured along mid-seam.

Straight plastron width (**SPW**): Straight-line distance measured at the level of the seam separating lower marginal scutes 6 and 7.

Curved plastron length (**CPL**): Curved distance from anterior to posterior plastron edge measured along mid-seam.

Curved plastron width (**CPW**): Curved distance measured at the level of the seam separating lower marginal scutes 6 and 7.

Medial seam length of plastral scutes (**GulL,** **HumL, PecL, AbdL, FemL, AnL**)

Width of anal fork (**AfW**): Distance from the tip of the anal scute to the other.

**Coloration related characters:**

Pigmentation of plastron in % (**Ppigm**)

Number of nasal stripes (**NasS**)

Number of ocular rings (**OkuR**)

Feature characteristics of ocular rings (**OkuRCharc**)

Shape of nuchale (**NuchSh**)

Coloration patterns of lower marginal scutes (**MargCol**)

Presence of chin stripe (**ChinS**)

Eye coloration (**EyeCol)**

Presence of connection of infraorbital stripe with crown (**InfLorbCon**)

Length of infraorbital stripe (**InfLorbL**)

Connection of infraorbital stripe to loreal seam broad or narrow (**InfLorb**)

Shape of infraorbital stripe (**InfLorbSh**)

Presence of postocular stripe (**postOcS**)

**S3.2.** Variable contribution to PCAs.

|  |  | **Females** | | | |  | **Males** | | | |
| --- | --- | --- | --- | --- | --- | --- | --- | --- | --- | --- |
| **Character** |  | **PC1** | **PC2** | **PC3** | **PC4** |  | **PC1** | **PC2** | **PC3** | **PC4** |
| SPL |  | 0.634 | 0.009 | 0.075 | 0.062 |  | 0.034 | 0.070 | 0.499 | 0.000 |
| CCL |  | 0.465 | 0.097 | 0.053 | 0.008 |  | 0.000 | 0.001 | 0.165 | 0.012 |
| SCW |  | 0.790 | 0.003 | 0.000 | 0.007 |  | 0.217 | 0.009 | 0.333 | 0.044 |
| CCW |  | 0.508 | 0.166 | 0.028 | 0.012 |  | 0.084 | 0.166 | 0.350 | 0.006 |
| HT |  | 0.397 | 0.012 | 0.013 | 0.005 |  | 0.000 | 0.374 | 0.205 | 0.037 |
| NL |  | 0.021 | 0.210 | 0.007 | 0.001 |  | 0.075 | 0.360 | 0.002 | 0.082 |
| NW |  | 0.002 | 0.172 | 0.169 | 0.022 |  | 0.238 | 0.064 | 0.054 | 0.024 |
| V1L |  | 0.261 | 0.029 | 0.066 | 0.013 |  | 0.000 | 0.112 | 0.053 | 0.232 |
| V1W |  | 0.112 | 0.439 | 0.014 | 0.019 |  | 0.431 | 0.001 | 0.005 | 0.006 |
| V2L |  | 0.266 | 0.133 | 0.196 | 0.119 |  | 0.053 | 0.036 | 0.037 | 0.257 |
| V2W |  | 0.442 | 0.208 | 0.001 | 0.018 |  | 0.196 | 0.065 | 0.222 | 0.011 |
| V3L |  | 0.091 | 0.147 | 0.039 | 0.256 |  | 0.038 | 0.031 | 0.067 | 0.002 |
| V3W |  | 0.484 | 0.202 | 0.053 | 0.003 |  | 0.238 | 0.122 | 0.185 | 0.042 |
| V4L |  | 0.197 | 0.044 | 0.175 | 0.073 |  | 0.077 | 0.002 | 0.010 | 0.494 |
| V4W |  | 0.411 | 0.231 | 0.006 | 0.035 |  | 0.018 | 0.043 | 0.175 | 0.000 |
| V5L |  | 0.114 | 0.019 | 0.117 | 0.274 |  | 0.019 | 0.007 | 0.004 | 0.588 |
| V5W |  | 0.102 | 0.064 | 0.007 | 0.119 |  | 0.201 | 0.001 | 0.013 | 0.316 |
| CPL |  | 0.662 | 0.001 | 0.075 | 0.035 |  | 0.063 | 0.091 | 0.184 | 0.029 |
| SPW |  | 0.591 | 0.120 | 0.002 | 0.020 |  | 0.227 | 0.005 | 0.500 | 0.001 |
| CPW |  | 0.660 | 0.169 | 0.008 | 0.000 |  | 0.186 | 0.004 | 0.328 | 0.004 |
| GulL |  | 0.052 | 0.191 | 0.146 | 0.201 |  | 0.123 | 0.021 | 0.000 | 0.107 |
| HumL |  | 0.265 | 0.211 | 0.000 | 0.205 |  | 0.019 | 0.069 | 0.127 | 0.240 |
| PecL |  | 0.001 | 0.230 | 0.250 | 0.003 |  | 0.191 | 0.608 | 0.016 | 0.000 |
| AbdL |  | 0.309 | 0.021 | 0.004 | 0.081 |  | 0.013 | 0.013 | 0.039 | 0.128 |
| FemL |  | 0.247 | 0.090 | 0.004 | 0.007 |  | 0.089 | 0.052 | 0.102 | 0.138 |
| AnL |  | 0.253 | 0.207 | 0.002 | 0.009 |  | 0.200 | 0.001 | 0.032 | 0.047 |
| AfW |  | 0.305 | 0.110 | 0.043 | 0.047 |  | 0.196 | 0.346 | 0.025 | 0.072 |
| Ppigm |  | 0.036 | 0.191 | 0.011 | 0.010 |  | 0.277 | 0.102 | 0.001 | 0.054 |
| NasS |  | 0.022 | 0.206 | 0.422 | 0.007 |  | 0.134 | 0.564 | 0.001 | 0.018 |
| OkuR |  | 0.018 | 0.323 | 0.135 | 0.048 |  | 0.315 | 0.211 | 0.003 | 0.140 |
| OkuRCharc |  | 0.227 | 0.238 | 0.803 | 0.124 |  | 0.656 | 0.875 | 0.045 | 0.101 |
| NuchSh |  | 0.102 | 0.241 | 0.178 | 0.262 |  | *NA* | *NA* | *NA* | *NA* |
| MargCol |  | *NA* | *NA* | *NA* | *NA* |  | 0.759 | 0.007 | 0.070 | 0.088 |
| ChinS |  | 0.014 | 0.041 | 0.677 | 0.004 |  | 0.117 | 0.344 | 0.027 | 0.001 |
| EyeCol |  | 0.164 | 0.381 | 0.153 | 0.225 |  | 0.479 | 0.032 | 0.047 | 0.125 |
| InfLorbCon |  | 0.010 | 0.103 | 0.601 | 0.019 |  | 0.158 | 0.400 | 0.002 | 0.002 |
| InfLorbL |  | 0.029 | 0.880 | 0.009 | 0.000 |  | 0.645 | 0.001 | 0.053 | 0.001 |
| InfLorb |  | 0.009 | 0.231 | 0.287 | 0.017 |  | 0.053 | 0.566 | 0.001 | 0.006 |
| InfLorbSh |  | 0.118 | 0.708 | 0.752 | 0.013 |  | 0.676 | 0.863 | 0.027 | 0.012 |
| postOcS |  | 0.050 | 0.573 | 0.034 | 0.008 |  | 0.279 | 0.150 | 0.095 | 0.018 |
